# IVEA: an integrative variational Bayesian inference method for predicting enhancer–gene regulatory interactions

**DOI:** 10.1093/bioadv/vbae118

**Published:** 2024-08-20

**Authors:** Yasumasa Kimura, Yoshimasa Ono, Kotoe Katayama, Seiya Imoto

**Affiliations:** DX Drug Discovery Department, Daiichi Sankyo RD Novare Co., Ltd., Edogawa-ku, Tokyo 134-8630, Japan; Division of Health Medical Intelligence, Human Genome Center, Institute of Medical Science, The University of Tokyo, Minato-ku, Tokyo 108-8639, Japan; Research Function Research Innovation Planning Department, Daiichi Sankyo Co., Ltd., Edogawa-ku, Tokyo 134-8630, Japan; DX Drug Discovery Department, Daiichi Sankyo RD Novare Co., Ltd., Edogawa-ku, Tokyo 134-8630, Japan; Division of Health Medical Intelligence, Human Genome Center, Institute of Medical Science, The University of Tokyo, Minato-ku, Tokyo 108-8639, Japan; Division of Health Medical Intelligence, Human Genome Center, Institute of Medical Science, The University of Tokyo, Minato-ku, Tokyo 108-8639, Japan

## Abstract

**Motivation:**

Enhancers play critical roles in cell-type-specific transcriptional control. Despite the identification of thousands of candidate enhancers, unravelling their regulatory relationships with their target genes remains challenging. Therefore, computational approaches are needed to accurately infer enhancer–gene regulatory relationships.

**Results:**

In this study, we propose a new method, IVEA, that predicts enhancer–gene regulatory interactions by estimating promoter and enhancer activities. Its statistical model is based on the gene regulatory mechanism of transcriptional bursting, which is characterized by burst size and frequency controlled by promoters and enhancers, respectively. Using transcriptional readouts, chromatin accessibility, and chromatin contact data as inputs, promoter and enhancer activities were estimated using variational Bayesian inference, and the contribution of each enhancer–promoter pair to target gene transcription was calculated. Our analysis demonstrates that the proposed method can achieve high prediction accuracy and provide biologically relevant enhancer–gene regulatory interactions.

**Availability and implementation:**

The IVEA code is available on GitHub at https://github.com/yasumasak/ivea. The publicly available datasets used in this study are described in Supplementary Table S4.

## 1 Introduction

Identification and characterization of regulatory elements (REs) responsible for regulating gene expression represent a central focus in modern genomics. More than 90% of the genomic variations associated with human traits and diseases are found in non-coding regions, many of which are thought to affect REs (Maurano *et al.* 2012). However, it is particularly difficult to identify relationships between enhancers located far from their regulatory target genes. Although recently developed detection methods, massively parallel reporter assays, and clustered regularly interspaced short palindromic repeats (CRISPR)-based methods have contributed extensively to the detection of regulatory relationships (Gasperini *et al.* 2020, Andersson and Sandelin 2020), the throughput of these approaches remains low. Computational predictions play an important role in characterizing these relationships.

Computational prediction of enhancer–gene relationships has been conducted using epigenetic features, such as histone modifications, chromatin openness and DNA methylation, protein–protein interactions, chromosomal conformation, and the expression of regulated genes. Some methods use a machine-learning approach with enhancer features (Whalen *et al.* 2016, Cao *et al.* 2017, Wang *et al.* 2021), whereas others rely on correlating enhancer features across multiple samples (Andersson *et al.* 2014, Duren *et al.* 2017, Silva *et al.* 2019). Several studies have combined various detection schemes (Fishilevich *et al.* 2017, Chen *et al.* 2018, Salviato *et al.* 2021). The recently proposed activity-by-contact (ABC) model (Fulco *et al.* 2019) and the generalized ABC model (Hecker *et al.* 2023) demonstrated high prediction accuracy when evaluated using CRISPR interference (CRISPRi) data. These models are based on the biochemical notion that an enhancer with higher activity and contact frequency with a gene promoter has a greater impact on gene expression. In these methods, the hypothetical ‘enhancer activity’ was determined by observed DNase-seq and/or H3K27ac ChIP-seq signals. However, obtaining H3K27ac ChIP-seq data is a laborious experiment. Although these signals have been shown to correlate with enhanced gene expression, enhancer activity is a hypothetical value that is influenced by other factors (Beagrie and Pombo 2016, Gasperini *et al.* 2020) and needs to be properly estimated. Since gene expression (RNA-seq) is typically obtained in all situations and represents the final outcome of gene regulation, we have chosen to utilize gene expression to estimate enhancer activity using a Bayesian approach.

In this study, our focus was on accurately estimating enhancer activities and leveraging them to predict enhancer–gene regulatory relationships. We built upon the understanding advanced in transcriptional bursting, which is a fundamental property of genes. Transcriptional bursting refers to the synthesis of transcripts in short discrete bursts, separated by longer periods of inactivity (Rodriguez and Larson 2020). The amount of transcripts is determined by the size of the bursts (burst size), the frequency of transitions to the active state (burst frequency), and the degradation rate of the transcripts. Recent research has shown that the control of transcriptional burst size and frequency lies with the promoter and enhancer, respectively (Bartman *et al.* 2016, Larsson *et al.* 2019). Using this characteristic in our statistical model, we developed a new method, IVEA, an *i*ntegrative *v*ariational Bayesian inference of regulatory *e*lement *a*ctivity for predicting enhancer–gene regulatory interactions. In this approach, gene expression is modelled by hypothetical promoter and enhancer activities, which reflect the regulatory potential of the promoters and enhancers, respectively. Using transcriptional readouts and functional genomic data of chromatin accessibility and contact as inputs, promoter and enhancer activities were estimated through variational Bayesian inference, and the contribution of each enhancer–promoter pair to target gene transcription was calculated. The enhancer–gene regulatory interactions predicted with the estimated enhancer activities reflected CRISPRi-based validation data and cis-eQTL data better than the other methods.

## 2 Methods

### 2.1 Methodological overview

**Figure 1.**
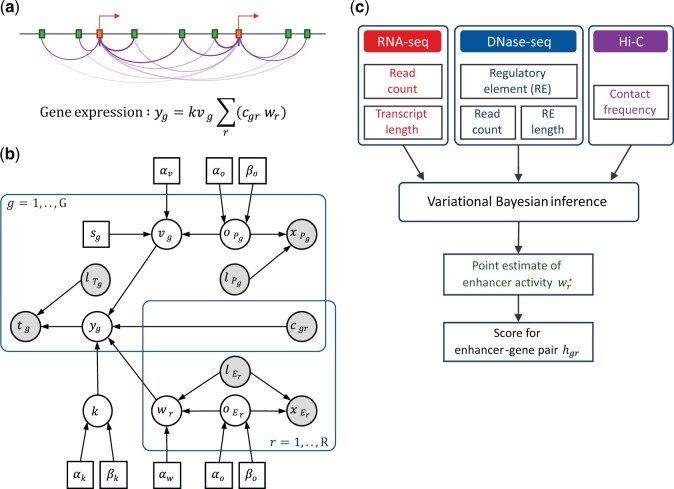
Methodological overview. (a) A conceptual representation of gene regulation by promoters and enhancers. Arrows indicate gene transcription start sites (TSSs). Boxes represent enhancer and promoter elements. Contacts between enhancer and promoter elements are depicted by arcs. The gene expression $y_{g}$ is expressed by promoter activity $v_{g}$, enhancer activity $w_{r}$, and their contact frequency $c_{gr}$. See also the notations in (b). (b) A graphical representation of the statistical model. Filled circles represent observed variables, unfilled circles represent latent variables, and boxes represent constant variables. For gene g, the RNA-seq read count, effective transcript length, gene expression, sequence-based burst size, promoter activity, promoter openness, promoter length, and promoter DNase-seq read count are represented by $t_{g},\;{l_{T}}_{g}$, $y_{g}$, $s_{g}$, $v_{g}$, ${o_{P}}_{g}$, ${l_{P}}_{g}$, and ${x_{P}}_{g}$, respectively. For enhancer r, activity, openness, length, and DNase-seq read count are represented by $w_{r}$, ${o_{E}}_{r}$, ${l_{E}}_{r}$, and ${x_{E}}_{r}$, respectively. Contact frequency between gene g and enhancer r is represented by $c_{gr}$. k is a scaling factor. (c) IVEA workflow.

Our model is based on transcriptional bursting (Bartman *et al.* 2016, Larsson *et al.* 2019) and ABC (Fulco *et al.* 2019) models. Transcriptional bursting characteristics reveal that transcriptional output is expressed as the product of burst size and frequency, which are highly dependent on promoters and enhancers, respectively (Bartman *et al.* 2016, Larsson *et al.* 2019). The ABC model calculates the enhancer regulatory strength as the product of enhancer activity and contact frequency with a target gene. Our proposed model combines these concepts. The contribution of each enhancer–promoter pair to target gene expression was expressed as the product of promoter activity, enhancer activity, and contact frequency. Multiple enhancers were assumed to contribute in an additive manner. Therefore, gene expression was expressed as the sum of multiple enhancer–promoter contributions (Fig. 1a). Using this as the core relationship, we constructed a Bayesian statistical model (Fig. 1b) in which gene expression, promoter/enhancer openness, and activity were treated as latent variables. For efficient computation, the approximate distributions of the latent variables were obtained using mean-field approximation and used for variational Bayesian inference.

Three datasets were used as inputs: chromatin accessibility (DNase-seq or ATAC-seq), gene expression (RNA-seq), and chromatin conformation (Hi-C). The REs were identified using DNase-seq (or ATAC-seq) peaks. REs detected in predefined promoter regions were used as promoter elements, and all detected REs were used as enhancer elements (Supplementary Fig. S1a). Read count, length features (calculated from DNase-seq, ATAC-seq, and RNA-seq), and contact frequency (calculated from Hi-C) were used as the observed data in the Bayesian model (Fig. 1c). Given a dataset, the promoter and enhancer activities were estimated using variational Bayesian inference based on the approximate distributions from the model. Finally, the contribution of each enhancer to a target gene was calculated and used to score enhancer–gene regulatory interactions (Fig. 1c).

### 2.2 Gene and transcription start site position

We used the curated RefSeq annotation provided in the ABC model (‘RefSeqCurated.170308. bed.CollapsedGeneBounds.bed’) to determine the gene positions. Briefly, the UCSC RefSeq track (refGene, version 2017-03-08) was downloaded, and each gene was linked to one transcription start site (TSS), where the TSS used by the largest number of coding isoforms was selected. Genes corresponding to small RNAs (gene symbol containing ‘MIR’ or ‘RNU’ or gene body length <300 bp) and very long RNAs (gene body >2 Mb) were removed. The positions for some genes (*FUT1*, *PVT1*, and *SMIM1*) were updated from ‘Supplementary Table 6a’ of the original paper (Fulco *et al.* 2019).

### 2.3 Promoter and enhancer elements

To identify REs, we followed the approach used in the ABC model. MACS2 (Zhang *et al.* 2008) was used to identify the peaks in DNase-seq (*P* < .1). The peaks overlapping blacklisted regions (‘wgEncodeHg19ConsensusSignalArtifactRegions.bed’) provided in ABC were removed. DNase-seq reads overlapping the detected peaks were counted, and the top 150 000 peaks with the highest number of mapped reads were retained. These peaks were resized to 500 bp and centred on the peak summit. Any overlapping regions resulting from peak extensions were merged. The extended and merged peaks were defined as the REs. The DNase-seq reads with overlapping REs were counted to determine their openness.

We selected regions from −1 kbp to +1 kbp of the TSSs as promoter regions. For simplicity, we established a promoter element for each gene. For multiple REs in one promoter region, the read counts and lengths were summed. Genes with no detected promoter elements were excluded from the analysis. We considered all REs as enhancer elements, including elements found in promoter regions, because gene promoters can potentially act as enhancers (Dao *et al.* 2017, Andersson and Sandelin 2020). If multiple REs were found in the promoter regions, the REs were treated individually as enhancers.

### 2.4 Chromatin contact frequency

The Knight-Ruiz (KR)-normalized Hi-C contact maps at 5-kb resolution were processed using the same procedure as for the ABC model: (i) Each diagonal entry of the Hi-C matrix was replaced by the maximum of its four neighbouring entries. (ii) All entries of the Hi-C matrix with a value of NaN or corresponding to KR normalization factors <0.25 were replaced with the expected contact under the power-law distribution. (iii) A small adjustment (pseudocount) was made to the contact frequency to ensure that the contact frequency for each enhancer–gene pair was nonzero.

We utilized both cell-type-specific Hi-C data (from K562 and GM12878 cells) (Rao *et al.* 2014) and the average of KR-normalized Hi-C contact maps from ten human cell types (‘ftp://ftp.broadinstitute.org/outgoing/lincRNA/average_hic/average_hic.v2.191020.tar.gz’). Since the K562 cell Hi-C data (Rao *et al.* 2014) lacked the KR-normalized 5-kb bin contact map of chromosome 9, vanilla-coverage (VC)-normalized (Lieberman-Aiden *et al.* 2009) data were used instead for chromosome 9.

### 2.5 Gene expression profile

Gencode annotation (‘gencode.v26lift37.annotation.gtf.gz’) was used as a reference for quantifying RNA-seq read counts. RNA-seq reads were aligned using STAR (Dobin *et al.* 2013) (v2.5.3a). Gene-level read counts and effective transcript lengths were determined using RSEM (v1.3.0) (Li and Dewey 2011).

To link the Gencode-based expression with the RefSeq-based gene positions described above, we used a two-step procedure: (i) Records whose gene symbols were matched between the curated RefSeq annotation and Gencode annotation were extracted. (ii) For the remaining records, those with positions that overlapped by >90% were linked. RefSeq records matched with non or multiple Gencode annotations were not used for the analysis.

### 2.6 Statistical model

The RNA-seq read count $t_{g}$ for gene *g* was modelled using a Poisson distribution:

(1) ptgyg=PoissontglTgyg,

where $y_{g}$ is the latent gene expression variable, and ${l_{T}}_{g}$ is the effective transcript length relative to 1000 nucleotides (nt). DNase-seq (or ATAC-seq) counts, ${x_{P}}_{g}\;$and ${x_{E}}_{r}$, in the promoter elements of gene *g* and enhancer element *r*, respectively, were also modelled using a Poisson distribution:

(2) pxPgoPg=PoissonxPglPgoPg,

(3) pxEroEr=PoissonxErlEroEr,

where ${o_{P}}_{g}\;$and ${o_{E}}_{r}$ are latent openness variables, and ${l_{P}}_{g}$ and ${l_{E}}_{r}$ are the element lengths relative to 1000 bp of the promoter and enhancer, respectively. These Poisson distributions are used to account for variations in observations. Although the openness variables do not directly appear in the equation of gene expression described below, considering them as latent variables improves prediction accuracy (Supplementary Table S1).

The latent gene expression variable $y_{g}$ was described using the transcriptional bursting mechanism, which is the predominant transcription mode (Panigrahi and O’Malley 2021). In the transcriptional bursting model, the transcriptional amount is expressed as the product of burst size and frequency (Bartman *et al.* 2016, Larsson *et al.* 2019). Following this concept, the gene expression $y_{g}$ can be written as

(4) yg=BSg· BFg,

where ${\mathrm{BS}}_{g}$ and ${\mathrm{BF}}_{g}$ are the burst size and burst frequency of gene *g* in a bulk sample, respectively. Larsson *et al* studied transcriptional burst kinetics in mammals in a single-cell setting and revealed that: (i) burst size can be estimated from the genomic sequence of the core promoter and the gene body length, (ii) burst frequencies have a strong linear dependence on enhancer activity, and (iii) incorporating gene-specific RNA half-lives has minor effects on estimating burst frequencies (Larsson *et al.* 2019). By performing a linear regression analysis for burst size, Larsson *et al* found that burst size $s_{g}$ could be estimated using DNA motifs residing in a core promoter and the gene body length (Larsson *et al.* 2019) (Supplementary Fig. S1b). As we modelled transcription in a bulk sample, the genomic sequence-based burst size $s_{g}$ was multiplied by the promoter openness ${o_{P}}_{g}$ as $s_{g}{o_{P}}_{g}$ in our model. However, the estimated $s_{g}$ has predictive variance, and the burst size is likely affected by *trans*-acting factors (Larsson *et al.* 2019). Considering these factors, we set a latent promoter activity $v_{g}$, the prior probability of which was modelled by a gamma distribution:

(5) pvgoPg=Gammavgαv, αvsgoPg,

where $\alpha_{v}$ is a hyperparameter that influences the variance (Supplementary Fig. S1c). Thus, the bulk burst size ${\mathrm{BS}}_{g}$ is expressed as $v_{g}$,

(6) BSg∝vg.

Although *v*_*g*_ is not directly related to enhancers, it plays a crucial role in formulating gene expression with enhancer–gene interactions. Omitting the promoter activity *v*_*g*_ resulted in a marked reduction in prediction accuracy (Supplementary Table S1).

Burst frequencies exhibit a strong linear dependence on H3K27ac enhancer signals (Larsson *et al.* 2019). Multiplying enhancer activity by contact frequency led to a successful prediction in the ABC model (Fulco *et al.* 2019). Most enhancers cooperate in an additive manner to regulate the transcription of target genes (Choi *et al.* 2021). Therefore, the bulk burst frequency ${\mathrm{BF}}_{g}$ in our model was expressed as the sum of the enhancer activities multiplied by their contact frequencies with a gene:

(7) BFg∝∑rcgrwr,

where $c_{gr}\;$is the contact frequency between gene g and enhancer r, and $w_{r}\;$is the latent enhancer activity of enhancer r. Enhancer elements located within a predefined distance (5 Mbp) from the gene TSS were analysed for their regulatory interactions. $c_{gr}\;$was set to zero if the distance between gene g and enhancer r was greater than a predefined distance.

Finally, the latent gene expression variable $y_{g}$ is described as

(8) yg=BSg·BFg=k·vg·∑rcgrwr,

where k is a scaling factor.

The enhancer activity $w_{r}$ was assumed to be related to chromatin openness, ${o_{E}}_{r}$, and element length, ${l_{E}}_{r}$, thereby reflecting the wide nucleosome-free regions commonly observed in active enhancers (Heinz *et al.* 2015, Li *et al.* 2016). However, enhancer activity is also affected by various regulatory factors that depend on enhancer sequence and cellular context (Heinz *et al.* 2015, Beagrie and Pombo 2016). Considering these factors, the prior probability of enhancer activity $w_{r}$ was modelled as:

(9) pwroEr=Gammawrαw, αwlEroEr,

where $\alpha_{w}$ is a hyperparameter that influences the variance (Supplementary Fig. S1c).

The prior distributions of openness ${o_{P}}_{g}$, ${o_{E}}_{r}$, and scaling factor k are also described by a gamma distribution:

(10) poPg=GammaoPg|αo,βo,

(11) poEr=GammaoEr|αo,βo,

(12) pk=Gammakαk,βk,

where $\alpha_{o}$, $\beta_{o}$, $\alpha_{k}$, and $\beta_{k}$ are hyperparameters of the gamma priors.

### 2.7 Variational Bayesian inference

Because the posterior distribution $p\left(Z|X\right)\;$where $Z=\left(v,w,o_{P},o_{E},k\right)$ and $X=(t,x_{P},x_{E},\;l_{T},l_{P},l_{E},C)$ does not have a closed-form solution, and because sampling methods are very time-consuming for actual data, such as those from the human genome, we performed variational Bayesian inference using a mean-field approximation (Jordan *et al.* 1999).

Suppose that q(Z) is a factorized distribution that approximates the posterior distribution $p\left(Z|X\right)$

(13) $$\begin{array}{c} q\left(Z\right)=q\left(v,w,o_{P},o_{E},k\right)=q\left(v\right)q\left(w\right)q\left(o_{P}\right)q\left(o_{E}\right)q\left(k\right)\\ =\prod_{g}q\left(v_{g}\right)\prod_{r}q\left(w_{r}\right)\prod_{g}q\left({o_{P}}_{g}\right)\prod_{r}q\left({o_{E}}_{r}\right)q\left(k\right) \end{array}.$$

The evidence lower bound (ELBO) on the log marginal likelihood ln⁡p(X) is described as

(14) $$\begin{array}{l} L(q)=E_{q}\left[\ln\;p\left(t|v,w,k\right)\right]+E_{q}\left[\ln\;p\left(x_{P}|o_{P}\right)\right]+E_{q}\left[\ln\;p\left(x_{E}|o_{E}\right)\right]\\ +E_{q}\left[\ln\;p\left(v|o_{P}\right)\right]+E_{q}\left[\ln\;p\left(w|o_{E}\right)\right]\\ \begin{array}{c} +E_{q}\left[\ln\;p\left(o_{P}\right)\right]+E_{q}\left[\ln\;p\left(o_{E}\right)\right]+E_{q}\left[\ln\;p\left(k\right)\right]\\ -E_{q}\left[\ln\;q\left(Z\right)\right],\; \end{array} \end{array}$$

where $E_{q}\left[.\right]$ represents the expectation under the distribution$\;q\left(Z\right)$. The optimization of $q\left(Z\right)$ was achieved by maximizing the ELBO L(q).

One issue here is that ELBO $L\left(q\right)$ contains an intractable expectation$\;E_{q}\left[\ln\left(\sum_{r}c_{gr}w_{r}\right)\right]$. Given the concavity of the natural logarithm, we introduced an auxiliary probability vector $\rho^{\left(g\right)}$ for gene g to the lower bound of this function following the procedure described in Paisley *et al.* (2014),

(15) ln∑rcgrwr≥∑rρrglncgrwr-∑rρrgln ρrg.

The expectation of the right side of Equation (15) is tractable. After each iteration of the variational inference algorithm, this auxiliary probability vector $\rho^{\left(g\right)}$ is optimized to obtain the tightest lower bound in the form

(16) ρrg=cgrexpEqln wr∑r′cgr′expEqln wr′.

By obtaining $q\left(Z\right)$ that maximizes $L\left(q\right)$, the form of distribution q for each of Z can be described by a generalized inverse Gaussian (GIG) distribution or gamma distribution:

(17) q*oPg=GIGoPgλPg,χPg, ψPg,

(18) q*oEr=GIGoErλEr,χEr,ψEr,

(19)  q*vg=Gammavgavg,bvg,

(20)  q*wr=Gammawrawr,bwr,

(21) q*k=Gammakak,bk,

where their parameters are described as

(22) λPg=αo-αv+xPg,

(23) χPg=2αvEqvgsg,

(24) ψPg=2βo+2lPg,

(25) λEr=αo-αw+xEr,

(26) χEr=2αwEqwrlEr,

(27) ψEr=2βo+2lEr,

(28) avg=αv+tg,

(29) bvg=αvsgEqoPg-1+lTgEqk∑rcgrEqwr,

(30) awr=αw+∑gtgρrg,

(31) bwr=αwlErEqoEr-1+Eqk∑glTgEqvg cgr,

(32) ak=αk+∑gtg,

(33) bk=βk+∑glTgEqvg∑rcgrEqwr.

The parameters and expectations were updated iteratively. The algorithm was stopped when all enhancer activities $w_{r}$ had a relative change of less than 5×10^−3^ or reached a maximum iteration of 500.

The hyperparameters are summarized in Supplementary Table S2, and the initial values of the random variables are summarized in Supplementary Table S3.

### 2.8 Enhancer–gene regulatory interaction score

After variational Bayesian inference, the promoter and enhancer activity point estimates are given by $v_{g}^{*}=E_{q}[v_{g}]$ and $w_{r}^{*}=E_{q}[w_{r}]$, respectively. The contribution of enhancer r to the expression of a particular gene g can be expressed as $v_{g}^{*}c_{gr}w_{r}^{*}$. The enhancer–gene regulatory interaction score $h_{gr}$ is calculated as its relative contribution to the expression of gene g and is written as

(34) hgr=vg*cgrwr*vg*∑r′cgr′ wr′* =cgrwr*∑r′cgr′ wr′* .

### 2.9 Promoter annotation for sequence-based burst size

To estimate the sequence-based burst size $s_{g}$ (Supplementary Fig. S1b), the presence or absence of TATA and Inr elements in each promoter region was retrieved from the eukaryotic promoter database (EPD) (Schmidt *et al.* 2021). The EPD is a non-redundant database of RNA polymerase II promoters with an experimentally validated TSS. Hs_EPDnew_005 and HsNC_EPDnew_001 were downloaded as protein-coding and non-coding genes, respectively. If a promoter region (±1 kbp from TSS) contained multiple EPD-defined promoters, the sequence-based burst size of each EPD promoter was estimated and averaged for the promoter region.

### 2.10 Evaluation data from CRISPRi perturbation

To evaluate predictive performance, we used datasets from three recent publications (Fulco *et al.* 2019, Gasperini *et al.* 2019, Schraivogel *et al.* 2020) that adopted CRISPRi perturbation. We focused on K562 cells, which were the most investigated cell types in the three datasets. We extracted positive and negative enhancer–gene pairs from each dataset based on the original authors’ analyses, as described below. The positive and negative enhancer–gene pairs from the three sets were combined and used for the analyses.

For the Fulco dataset, we used ‘Supplementary Table 6a’ from the original paper (Fulco *et al.* 2019). From the complete list of examined non-coding element-gene pairs, those pairs involving non-coding elements classified as ‘promoter’ were removed. Following the original authors’ approach, we used the pairs where the ‘Significant’ field is ‘TRUE’ and the ‘Fraction change in gene expr’ field <0 as positive enhancer–gene pairs and those with the ‘Significant’ field as ‘FALSE’ or the ‘Fraction change in gene expr’ field ≥0 as negative enhancer–gene pairs. This resulted in 109 positive and 3754 negative enhancer–gene pairs.

For the Gasperini dataset, we downloaded the complete list of gRNA-target gene pairs of the scaled multiplex enhancer–gene pair screen from the Gene Expression Omnibus repository (‘GSE120861_all_deg_results.at_scale.txt.gz’) and the enhancer–gene pairs list ‘Table S2B’ from the supplementary materials of the original paper (Gasperini *et al.* 2019). The original authors defined two sets of enhancer–gene pairs, the inclusive set (all the 664 pairs in ‘Table S2B’) and the high confidence set (470 pairs with the high_confidence_subset field is ‘TRUE’ in ‘Table S2B’). The inclusive enhancer–gene pairs only required a 10% empirical FDR in the scaled experiment, whereas the high-confidence enhancer–gene pairs required reproducible results in both the pilot and scaled experiments or were internally reproducible between the two independent gRNAs. We used the 470 high-confidence enhancer–gene pairs as positive enhancer–gene pairs. For the negative enhancer–gene pairs, we started with all examined enhancer–gene pairs in ‘GSE120861_all_deg_results.at_scale.txt.gz’, except for those classified as promoter proximal elements (‘TSS’ and ‘selfTSS’) or positive and negative controls (‘NTC’ and ‘positive_ctrl’). We removed 664 inclusive enhancer–gene pairs from all examined pairs and retained pairs whose genes appeared in the high-confidence set, resulting in 3458 negative enhancer–gene pairs.

For the Schraivogel dataset, we used the differential expression results ‘http://steinmetzlab.embl.de/TAPdata/diff_expr_screen_nGenesCovar.csv’ produced in their study. From the entire list of examined pairs, we extracted cis enhancer–gene pairs where the ‘enh_type’ field is ‘cis’, the ‘perturbation’ field is not a gene name, and the pair distance is between 1 kb and 5 Mb. We converted the genomic coordinates of the candidate enhancers from hg38 to hg19 using the UCSC liftOver tool. We considered pairs where the ‘pval_adj_allTests’ (FDR) field is <0.05, the ‘logFC’ field is <0, and the ‘grna_hits’ field is >0 as positive enhancer–gene pairs. For the negative enhancer–gene pairs, we kept pairs whose genes appeared in the positive enhancer–gene pairs and extracted the pairs with a ‘logFC’ field >0. This resulted in 37 positive and 3930 negative enhancer–gene pairs.

### 2.11 Enhancer–gene pairs identified by other methods

To benchmark our IVEA method, we compared its performance with ABC (Fulco *et al.* 2019) (v0.2.2), STARE (Hecker *et al.* 2023), TargetFinder (Whalen *et al.* 2016), JEME (Cao *et al.* 2017), and ProTECT (Wang *et al.* 2021) which provide cell-type-specific predictions.

In the ABC model, both DNase-seq and H3K27ac ChIP-seq data were used for enhancer activity, whereas IVEA used only DNase-seq data as input. For comparison, we performed two types of ABC predictions: the original ABC model using the geometric mean of DNase-seq and H3K27ac ChIP-seq data (ABC–H3K27ac) and the ABC model using only DNase-seq data (ABC–DHS). Predictions were made using the data on K562 and GM12878 cells in this study (Supplementary Table S4) with default parameters. The ‘ABC.Score’ was used to score the predicted enhancer–gene pairs.

Hecker *et al* proposed two types of refinements for the ABC scores implemented in STARE. Firstly, they considered enhancer activity in a gene-specific manner by proportionally allocating relative activity to each target gene according to its contact. Secondly, they used the information of all annotated TSSs of a gene, enabling the inclusion of contact information for more potentially relevant transcription sites. For comparison, we conducted two types of scoring schemes: the generalized ABC (gABC) score, which considers these two refinements (STARE_ABCpp -i all_tss -q True), and the ABC score considering all annotated TSSs (ABC_TSS) (only the second refinement) (STARE_ABCpp -i all_tss -q False). Regarding enhancer activity, we applied different types of activities: the geometric mean of DNase-seq and H3K27ac ChIP-seq data (–H3K27ac), only DNase-seq (–DHS), and estimated activities from IVEA using gene expression >8 transcripts per million (TPM) (–IVEA). The predictions were made using the data on K562 and GM12878 cells in this study (Supplementary Table S4) with a Gencode annotation (-a gencode.v26lift37.annotation.gtf), a window size of ±5 Mb from TSS (-w 10000000), and displaying all scores (-t 0). The ‘ABC-Score’ was used to score the predicted enhancer–gene pairs.

For TargetFinder, the predictions for K562 and GM12878 cells were downloaded from https://github.com/shwhalen/targetfinder. We used the predictions generated by the gradient-boosting classifier for the enhancer, promoter, and window regions. Pairs of enhancer and promoter regions (no gene IDs provided) were used for evaluation. The ‘prediction’ field was used as a pair score.

For JEME, lasso-based predictions for K562 (ID 121) and GM12878 (ID 114) cells were downloaded from http://yiplab.cse.cuhk.edu.hk/jeme/. Because gene TSSs can differ according to the original annotations, enhancer position–gene symbol pairs were used for evaluation. The third field was used as a pair score.

For ProTECT, the predictions for K562 and GM12878 cells were downloaded from https://github.com/wangjr03/PPI-based_prediction_enh_gene_links. Because gene TSSs can differ according to the original annotations, the enhancer position–Ensembl gene stable ID pairs were used for evaluation. The seventh field was used as a pair score.

### 2.12 Evaluation with cis-eQTL data

We also evaluated the predictive performance using cis-eQTLs by checking their overlap with the predicted enhancer–gene pairs. We used the cis-eQTL datasets of whole blood in GTEx (Aguet *et al.* 2017) and lymphoblastoid cell lines (LCLs) in GEUVADIS (Lappalainen *et al.* 2013). For the GTEx dataset, GTEx v8 single-tissue cis-eQTL data (‘GTEx_Analysis_v8_eQTL.tar’) were downloaded from https://gtexportal.org/home/datasets. We used all significant variant-gene pairs in whole blood identified in GTEx (Whole_Blood.v8.signif_variant_gene_pairs.txt.gz). The variant positions were converted into hg19 using the UCSC liftOver tool, and gene symbols were set using the annotation in the release (‘Whole_Blood.v8.egenes.txt.gz’). For the GEUVADIS dataset, the cis-eQTL results of LCLs from 373 individuals of European descent (EUR) and 89 Yoruban individuals (YRI) were downloaded from http://ftp.ebi.ac.uk/pub/databases/microarray/data/experiment/GEUV/E-GEUV-1/analysis_results/ (‘EUR373.gene.cis.FDR5.all.rs137.txt.gz’; ‘YRI89.gene.cis.FDR5.all.rs137.txt.gz’). Because the gene identifiers were based on Gencode v12, gene symbols were retrieved from the Gencode v12 annotation (‘gencode.v12.annotation.gtf.gz’).

Because both cis-eQTL datasets comprised variants located within 1 Mb of their target gene TSSs, we limited the evaluated enhancer–gene pairs to a distance from 1 kb to 1 Mb. A predicted enhancer–gene pair was considered eQTL-supported if the enhancer–gene pair overlapped with at least one variant-gene pair.

### 2.13 Characterization of predicted enhancers by transcription factor binding motifs

Enhancers are regulated by bound transcription factors (TFs); numerous TFs have been characterized by specific biological functions. To characterize the predicted enhancer–gene interactions, we investigated TF-binding motifs in the predicted enhancers. We extracted TF footprints from the enhancer regions and identified TF motifs within the footprints using RGT (Li *et al.* 2023). TF footprints were analysed using rgt-hint (footprinting --organism hg19 --paired-end --dnase-seq --bias-correction). From the extracted footprint regions, TF binding motifs were detected using rgt-motifanalysis (matching --organism hg19) using HOCOMOCOv11 full mononucleotide models as a motif database. To focus on the active TFs in a particular cell type, the detected motifs were filtered by the expression of the corresponding TF genes to have at least two TPM. The number of detected binding motifs for each TF gene was compared between different prediction methods using the chi-square test. The *P* values were corrected using the false discovery rate *q*-values estimated using the positive false discovery rate method (Storey 2003).

### 2.14 Analysis of genome-wide association studies variants

Many disease-associated SNPs are located in non-coding loci and may affect enhancers. One important application of enhancer–gene interaction prediction is to map genome-wide association studies (GWAS) variants to target genes through enhancers. To investigate the applicability of our method, we analysed autoimmune disease GWAS variants in GM12878 LCL. Autoimmune disease GWAS data with child traits (EFO ID: EFO_0005140) were downloaded from the NHGRI-EBI GWAS catalogue (Sollis *et al.* 2023) (https://www.ebi.ac.uk/gwas/) on 22 August 2023. Variants with multiple or no available genomic positions were excluded from the analysis. The variant positions in hg38 were converted to positions in hg19 using the UCSC liftOver tool. Given a set of predicted enhancer–gene pairs, enhancers containing GWAS variants were selected. Target genes predicted to be regulated by variant-containing enhancers were then extracted. Gene ontology enrichment of the extracted variant-linked genes was analysed using GOrilla (Eden *et al.* 2009) (https://cbl-gorilla.cs.technion.ac.il/, with the database updated on 6 March 2021**)** using a list of all genes in the given set of predicted pairs as a background.

## 3 Results

### 3.1 Effect of hyperparameters

**Figure 2.**
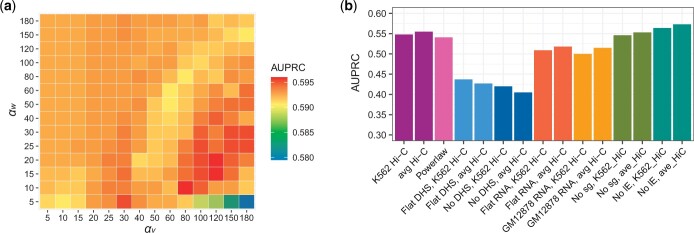
Effects of hyperparameters and input data evaluated using CRISPRi perturbation data. IVEA was performed with an 8 TPM cut-off. (a) AUPRCs of IVEA predictions with different hyperparameters $\alpha_{v}$ and $\alpha_{w}$ in the K562 training data; the values are averaged between the use of K562 Hi-C and the average Hi-C matrix. (b) AUPRCs of IVEA with different settings of each input data in the K562 test data. ‘K562 Hi-C’, ‘avg Hi-C’, and ‘Powerlaw’ denote the use of the K562 Hi-C, the averaged Hi-C matrix, and a power-law function of distance ($\mathrm{Contact}\;\approx {\mathrm{Distance}}^{-0.7}$) for chromatin contact information, respectively. ‘Flat DHS’ and ‘No DHS’ refer to the use of a flat read count (1000) across all REs and the exclusion of DHS values from the model, respectively. ‘Flat RNA’ and ‘GM12878 RNA’ denote the application of an artificial flat RNA-seq read count (1000) across all genes and incompatible RNA expression values from GM12878 cells, respectively. ‘No sg’ and ‘No lE’ refer to the model omitting the sequence-based burst size $s_{g}$ in the prior information of promoter activity and omitting the relative enhancer element length ${l_{E}}_{r}$ in the prior information of enhancer activity, respectively. AUPRC = area under the precision–recall curve.

To evaluate IVEA, we first analysed K562 cells (the dataset used is summarized in Supplementary Table S4), for which a large number of enhancer–gene pairs validated by CRISPRi perturbation were available. We combined K562 CRISPRi data from three publications (Fulco *et al.* 2019, Gasperini *et al.* 2019, Schraivogel *et al.* 2020) in which there were only a few shared enhancer–gene pairs among the studies (Supplementary Fig. S2). The CRISPRi data were divided into training and test sets by chromosomes (Supplementary Table S5), and the training data were used to optimize the hyperparameters. The optimized hyperparameters were the shape parameters $\alpha_{v}$ and $\alpha_{w}$ in the gamma priors of the promoter and enhancer activity, respectively, which determine the degree of variation in their prior distributions (Supplementary Fig. S1c). In the optimization, genes with expression >8 TPM were analysed using two sets of Hi-C matrix: the K562 Hi-C matrix and the average Hi-C matrix. Both $\alpha_{v}$ and $\alpha_{w}$ varied from 5 to 180 in the prediction against the training data. The area under the precision–recall curve (AUPRC) for each prediction was calculated using the CRISPRi-validated enhancer–gene pair dataset (Supplementary Fig. S3). Subsequently, the two sets of AUPRCs obtained using the K562 Hi-C matrix and the average Hi-C matrix were averaged (Fig. 2a). Among the examined hyperparameter values, the combination of hyperparameters that provided the highest AUPRC ($\alpha_{v}$= 80 and $\alpha_{w}$= 10) was selected as the optimal one and applied for the subsequent analyses. With the optimal values, the prior distribution of the enhancer activity had a larger coefficient of variation than that of the promoter activity.

### 3.2 Effect of input data

First, we examined the effect of the data of each input by removing or changing its values (Fig. 2b). We evaluated the effects using AUPRCs, which were calculated using the K562 test dataset of CRISPRi-validated enhancer–gene pairs. We set the AUPRC of IVEA using the K562 Hi-C matrix and genes expressed >8 TPM as a baseline. Replacing the K562 Hi-C matrix with the averaged Hi-C matrix resulted in a slight increase of 0.007, while using a power-law function of distance ($\mathrm{Contact}\;\approx {\mathrm{Distance}}^{-0.7}$) (Supplementary Fig. S4a) resulted in a slight decrease of −0.007 in AUPRC (Fig. 2b). Although contact information between chromatin regions was the most crucial factor (Supplementary Fig. S4b), the Hi-C matrix can be substituted with either the averaged Hi-C matrix or the power-law function of distance if acquiring cell-type-specific contact information proves challenging. Changing the DHS values to a flat read count of 1000 across all REs (Flat DHS) resulted in a decrease of −0.111. Excluding the DHS values from the model (No DHS) resulted in an additional decrease of −0.128 (Fig. 2b). This suggests that using observed chromatin accessibility is necessary for accurate prediction. These reductions in performance due to DHS values were more pronounced when using the averaged Hi-C matrix. IVEA using an artificial flat RNA-seq read count (1000) over all genes (Flat RNA) caused a loss of −0.039, and using incompatible RNA expression values from GM12878 cells (GM12878 RNA) caused a further loss of −0.048 (Fig. 2b). This suggests the effectiveness of using cell-type-specific gene expressions. We also examined the effect of the sequence-based burst size $s_{g}$ in Equation (5) and the relative enhancer element length ${l_{E}}_{r}$ in Equation (9). When omitting $s_{g}$ in the prior information of promoter activity (No sg), a slight decrease of −0.002 in AUPRC was observed. On the other hand, the model omitting ${l_{E}}_{r}$ in the prior information of enhancer activity (No lE) (hereafter denoted as ‘IVEA_nolE’, details in Supplementary Note S1) showed an increase of 0.016 in AUPRC (Fig. 2b).

### 3.3 Effect of gene expression levels on inference

Since enhancers are responsible for gene activation, it is necessary for the target genes in enhancer–gene interaction analysis to be expressed. Therefore, we examined the appropriate expression levels of the target genes in the IVEA inference. We studied the impact of the expression cut-off by changing it to 0, 1, 2, 4, 8, 16, and 32 TPM in the K562 test dataset. The number of analysed genes decreased as the cut-off increased (Supplementary Table S6). To accommodate for the variation in analysed genes, the evaluations were performed against different sets of CRISPRi validated enhancer–gene pairs, filtered by the expression levels of the target genes (>0, >1, >2, >4, >8, >16, and >32 TPM) (Supplementary Table S6). When the expression cut-off was set to 0, many genes, including very lowly expressed genes, could be analysed, but the AUPRCs were lower compared to those with higher cut-offs for IVEA (Table 1). When a higher expression cut-off was set for IVEA, higher AUPRCs were observed (Table 1). However, this trend was observed up to 8 TPM. When the cut-off was 16 TPM, the AUPRC was slightly lower than those with 4 and 8 TPM cut-offs (Table 1). These results suggest that IVEA provides high accuracy using a cut-off from 4 to 8 TPM. A similar trend was observed for IVEA_nolE, but the AUPRC was also high at a 16 TPM cut-off (Supplementary Table S7). For genes with very low expression, the enhancer contributions should be negligible, or silencers could contribute, which might reduce the prediction accuracy by increasing noise. Conversely, if the cut-off is very high, the prediction accuracy may decrease because of insufficient input data.

**Table 1.** AUPRCs of IVEA with different expression cut-offs evaluated using K562 CRISPRi test data.^a^

	IVEA gene expression cut-off [TPM]	Expression levels of evaluated genes [TPM]						
		>0	>1	>2	>4	>8	>16	>32
K562 Hi-C	IVEA cut-off 0	**0.523**	0.525	0.53	0.529	0.538	0.555	0.566
	IVEA cut-off 1		**0.531**	0.536	0.534	0.541	0.558	0.569
	IVEA cut-off 2			**0.54**	0.539	0.545	0.562	0.572
	IVEA cut-off 4				**0.542**	**0.548**	0.565	**0.576**
	IVEA cut-off 8					**0.548**	**0.566**	0.574
	IVEA cut-off 16						0.565	0.571
	IVEA cut-off 32							0.568
avg. Hi-C	IVEA cut-off 0	**0.528**	0.531	0.536	0.535	0.542	0.557	0.558
	IVEA cut-off 1		**0.54**	0.545	0.544	0.549	0.564	0.57
	IVEA cut-off 2			**0.549**	0.548	0.553	0.568	0.575
	IVEA cut-off 4				**0.55**	**0.555**	0.57	0.576
	IVEA cut-off 8					**0.555**	**0.571**	**0.577**
	IVEA cut-off 16						0.568	0.573
	IVEA cut-off 32							0.565

a The highest AUPRC value within each column and type of Hi-C data is highlighted in bold.

Abbreviations: AUPRC = area under the precision–recall curve; TPM = transcripts per million.

We also examined an IVEA model that analyses all annotated transcriptional isoforms to handle all TSSs. However, the evaluation with expression cut-offs showed that the AUPRCs were lower than those from the gene-based IVEA model, especially at the low expression cut-offs (Supplementary Table S8). The inclusion of lowly expressed transcriptional isoforms in the inference presumably caused the lower prediction accuracy.

### 3.4 Comparison with other prediction methods

To assess the performance of IVEA, we compared its prediction performance with other methods: original ABC (Fulco *et al.* 2019) (ABC), ABC considering all TSSs (Hecker *et al.* 2023) (ABC_TSS), generalized ABC (Hecker *et al.* 2023) (gABC), JEME (Cao *et al.* 2017), ProTECT (Wang *et al.* 2021), and TargetFinder (Whalen *et al.* 2016). ABC, ABC_TSS, and gABC were evaluated with four types of enhancer activities: the geometric mean of H3K27ac and DHS signals (–H3K27ac), DHS signals only (–DHS), IVEA-estimated activities (–IVEA), and IVEA_nolE-estimated activities (–IVEA_nolE).

We initially evaluated each method using the CRISPRi-validated enhancer–gene pairs in the K562 test dataset. To evaluate the predictions that vary based on the expression cut-off applied to IVEA, we conducted comparisons using different sets of validated pairs filtered by expression levels of target genes (>0, >1, >2, >4, >8, >16, and >32 TPM). When we evaluated each method using validated pairs of genes expressed at >4, >8, >16, and >32 TPM, IVEA_nolE, and IVEA showed high AUPRCs for each evaluation pair set (Table 2). It is important to note that when evaluating the methods using genes expressed at >16 and >32 TPM, the AUPRCs were even higher for IVEA using a 4 and 8 TPM cut-off in the inference, as shown in Table 1. However, we included the results from the same cut-offs as the evaluated genes in Table 2. When evaluating each method using validated pairs of genes with TPM values >0, >1, and >2, the ABC_TSS–IVEA_nolE and ABC_TSS–IVEA methods, which consider all TSSs in ABC with IVEA_nolE- and IVEA-estimated enhancer activities, demonstrated high accuracy (Table 2). This suggests that enhancer activities estimated by IVEA can contribute to predicting enhancer–gene interactions for genes with low expression levels. The use of enhancer activities estimated from IVEA_nolE (–IVEA_nolE) instead of the original IVEA (–IVEA) showed higher AUPCRC values in the ABC and ABC_TSS scoring schemes, with a tendency for larger differences in AUPRCs at higher TPM thresholds. However, in gABC, gABC–IVEA_nolE, and gABC–IVEA showed similar AUPRCs (Table 2). As in a previous study (Hecker *et al.* 2023), the results using only DHS (–DHS) and both DHS and H3K27ac (–H3K27ac) were comparable, with DHS only (–DHS) performing better in the Gasperini dataset (Supplementary Fig. S5).

**Table 2.** AUPRCs of each method evaluated using the CRISPRi-validated enhancer–gene pairs with varying expression levels of target genes in the K562 test dataset.^a^

	Method	Expression levels of evaluated genes [TPM]						
		>0	>1	>2	>4	>8	>16	>32
K562 Hi-C	ABC–H3K27ac	0.518	0.527	0.53	0.526	0.521	0.547	0.556
	ABC–DHS	0.532	0.534	0.538	0.534	0.533	0.55	0.554
	IVEA	0.523	0.531	0.54	0.542	0.548	0.565	0.568
	IVEA_nolE	0.53	0.541	0.55	**0.555**	**0.564**	**0.587**	**0.588**
	ABC_TSS–H3K27ac	0.523	0.533	0.537	0.531	0.529	0.548	0.546
	ABC_TSS–DHS	0.531	0.533	0.538	0.534	0.536	0.551	0.544
	ABC_TSS–IVEA	0.54	0.542	0.546	0.541	0.546	0.561	0.556
	ABC_TSS–IVEA_nolE	**0.545**	**0.547**	**0.552**	0.548	0.559	0.576	0.568
	gABC–H3K27ac	0.531	0.54	0.546	0.539	0.539	0.561	0.566
	gABC–DHS	0.535	0.535	0.54	0.534	0.539	0.557	0.553
	gABC–IVEA	0.531	0.532	0.535	0.529	0.533	0.55	0.548
	gABC–IVEA_nolE	0.53	0.529	0.533	0.528	0.537	0.553	0.547
avg. Hi-C	ABC–H3K27ac	0.513	0.526	0.529	0.525	0.519	0.545	0.554
	ABC–DHS	0.528	0.533	0.538	0.535	0.532	0.55	0.555
	IVEA	0.528	0.54	0.549	0.55	0.555	0.568	0.565
	IVEA_nolE	0.54	0.548	0.559	**0.563**	**0.573**	**0.59**	**0.587**
	ABC_TSS–H3K27ac	0.52	0.531	0.536	0.53	0.526	0.544	0.544
	ABC_TSS–DHS	0.53	0.535	0.54	0.536	0.536	0.549	0.544
	ABC_TSS–IVEA	0.543	0.547	0.551	0.547	0.549	0.562	0.556
	ABC_TSS–IVEA_nolE	**0.554**	**0.556**	**0.561**	0.558	0.566	0.58	0.569
	gABC–H3K27ac	0.526	0.534	0.542	0.536	0.535	0.554	0.557
	gABC–DHS	0.529	0.53	0.536	0.529	0.532	0.549	0.542
	gABC–IVEA	0.523	0.524	0.528	0.522	0.524	0.538	0.533
	gABC–IVEA_nolE	0.525	0.525	0.528	0.523	0.53	0.544	0.536
Hi-C-indep.	JEME	0.0517	0.0517	0.0493	0.0448	0.0354	0.0335	0.0339
	ProTECT	0.00879	0.00879	0.00879	0.0089	0.00904	0.0081	0.00698
	TargetFinder	0.0121	0.0123	0.0123	0.0104	0.0115	0.0108	0.00818

a The cut-off in IVEA and IVEA_nolE inferences was set to be the same as that for the evaluated genes. The highest AUPRC within each column and type of Hi-C data is highlighted in bold.

Abbreviations: AUPRC = area under the precision–recall curve; TPM = transcripts per million.

Next, we evaluated prediction performance using cis-eQTL datasets. Here, we used the expression cut-off of 8 TPM and expanded our analysis to GM12878 cells (the dataset used is summarized in Supplementary Table S4) in addition to K562 cells. The cis-eQTL datasets of whole blood in GTEx (Aguet *et al.* 2017) and LCLs in GEUVADIS (Lappalainen *et al.* 2013) were used for the evaluation. To evaluate, we focused on a set of top 10 high-scored enhancer–gene pairs for each gene (hereafter denoted as ‘top-10-pair set’) and examined overlaps with the eQTLs. The number of eQTL-supported enhancer–gene pairs from the different approaches is shown in Table 3. In K562 cells, approaches that utilize IVEA-estimated enhancer activities predicted a greater number of eQTL-supported enhancer–gene pairs compared to those that utilize other activity values (–IVEA versus –H3K27ac and –IVEA versus –DHS in ABC, ABC_TSS, and gABC) in both the GTEx whole blood and GEUVADIS LCLs datasets. IVEA_nolE-estimated enhancer activities (–IVEA_nolE) further increased the number of eQTL-supported enhancer–gene pairs in ABC and ABC_TSS but not in gABC. This trend remained consistent when using the K562 Hi-C matrix and the average Hi-C matrix. Among these approaches, gABC using IVEA-estimated enhancer activities (gABC–IVEA) predicted the highest number of eQTL-supported enhancer–gene pairs (4853 and 929 in the GTEx and GEUVADIS datasets with the K562 Hi-C, respectively, and 4914 and 940 in the GTEx and GEUVADIS datasets with the average Hi-C, respectively). In GM12878 cells, a similar trend as in K562 cells was observed when using both the average Hi-C matrix and the GM12878 Hi-C matrix (Table 3). Approaches utilizing IVEA-estimated enhancer activities (–IVEA) predicted a larger number of eQTL-supported enhancer–gene pairs compared to those utilizing other activity values (–H3K27ac and –DHS). IVEA_nolE-estimated enhancer activities (–IVEA_nolE) further increased the number of eQTL-supported enhancer–gene pairs in ABC and ABC_TSS but not in gABC. gABC–IVEA predicted the largest number of eQTL-supported enhancer–gene pairs (11 931 and 2590 in the GTEx and GEUVADIS datasets with the GM12878 Hi-C, respectively, and 11 981 and 2561 in the GTEx and GEUVADIS datasets with the average Hi-C, respectively). When analysing a set of the top five enhancer–gene pairs for each gene, a similar tendency to the top-10-pair set was observed (Supplementary Table S9).

**Table 3.** The number of eQTL-supported enhancer–gene pairs in the top-10-pair set.^a^

	Method	K562			GM12878		
		Number of predicted pairs	Number of eQTL-supported pairs (% in predicted pairs)		Number of predicted pairs	Number of eQTL-supported pairs (% in predicted pairs)	
			GTEx Whole blood	GEUVADIS LCLs		GTEx Whole blood	GEUVADIS LCLs
Cell-type-specific Hi-C	ABC–H3K27ac	34 662	3471 (10.01%)	634 (1.83%)	79 900	8618 (10.79%)	1836 (2.30%)
	ABC–DHS	34 662	3470 (10.01%)	630 (1.82%)	79 900	8576 (10.73%)	1834 (2.30%)
	IVEA	34 638	3715 (10.73%)	684 (1.97%)	79 894	9079 (11.36%)	1942 (2.43%)
	IVEA_nolE	34 638	3739 (10.79%)	702 (2.03%)	79 894	9317 (11.66%)	2017 (2.52%)
	ABC_TSS–H3K27ac	34 589	3766 (10.89%)	701 (2.03%)	79 860	9561 (11.97%)	2063 (2.58%)
	ABC_TSS–DHS	34 608	3798 (10.97%)	696 (2.01%)	79 864	9587 (12.00%)	2049 (2.57%)
	ABC_TSS–IVEA	34 608	4052 (11.71%)	749 (2.16%)	79 864	10 086 (12.63%)	2177 (2.73%)
	ABC_TSS–IVEA_nolE	34 608	4113 (11.88%)	788 (2.28%)	79 864	10 348 (12.96%)	2248 (2.81%)
	gABC–H3K27ac	34 589	4701 (13.59%)	898 (2.60%)	79 860	11 683 (14.63%)	2527 (3.16%)
	gABC–DHS	34 608	4781 (13.81%)	900 (2.60%)	79 864	11 845 (14.83%)	2553 (3.20%)
	gABC–IVEA	34 608	**4853** (14.02%)	**929** (2.68%)	79 864	**11** **931** (14.94%)	**2590** (3.24%)
	gABC–IVEA_nolE	34 608	4836 (13.97%)	926 (2.68%)	79 864	11 901 (14.90%)	2581 (3.23%)
avg. Hi-C	ABC–H3K27ac	34 662	3410 (9.84%)	622 (1.79%)	79 900	8529 (10.67%)	1831 (2.29%)
	ABC–DHS	34 662	3395 (9.79%)	620 (1.79%)	79 900	8464 (10.59%)	1782 (2.23%)
	IVEA	34 638	3651 (10.54%)	679 (1.96%)	79 894	8960 (11.21%)	1922 (2.41%)
	IVEA_nolE	34 638	3723 (10.75%)	695 (2.01%)	79 894	9214 (11.53%)	1976 (2.47%)
	ABC_TSS–H3K27ac	34 589	3786 (10.95%)	699 (2.02%)	79 860	9564 (11.98%)	2046 (2.56%)
	ABC_TSS–DHS	34 608	3822 (11.04%)	701 (2.03%)	79 864	9624 (12.05%)	2030 (2.54%)
	ABC_TSS–IVEA	34 608	4076 (11.78%)	762 (2.20%)	79 864	10 106 (12.65%)	2162 (2.71%)
	ABC_TSS–IVEA_nolE	34 608	4168 (12.04%)	788 (2.28%)	79 864	10 344 (12.95%)	2239 (2.80%)
	gABC–H3K27ac	34 589	4771 (13.79%)	901 (2.60%)	79 860	11 696 (14.65%)	2536 (3.18%)
	gABC–DHS	34 608	4835 (13.97%)	929 (2.68%)	79 864	11 847 (14.83%)	2546 (3.19%)
	gABC–IVEA	34 608	**4914** (14.20%)	**940** (2.72%)	79 864	**11** **981** (15.00%)	**2561** (3.21%)
	gABC–IVEA_nolE	34 608	4901 (14.16%)	939 (2.71%)	79 864	11 879 (14.87%)	2551 (3.19%)
Hi-C-indep.	JEME	12 830	1851 (14.43%)	329 (2.56%)	26 436	4058 (15.35%)	926 (3.50%)
	ProTECT	18 017	1786 (9.91%)	277 (1.54%)	33 194	2205 (6.64%)	450 (1.36%)
	TargetFinder	12 453	249 (2.00%)	39 (0.31%)	29 238	865 (2.96%)	167 (0.57%)

a The highest number of pairs within each eQTL dataset and type of Hi-C data is highlighted in bold.

Abbreviation: LCLs = lymphoblastoid cell lines.

### 3.5 Characteristics of predicted enhancer–gene interactions

**Figure 3.**
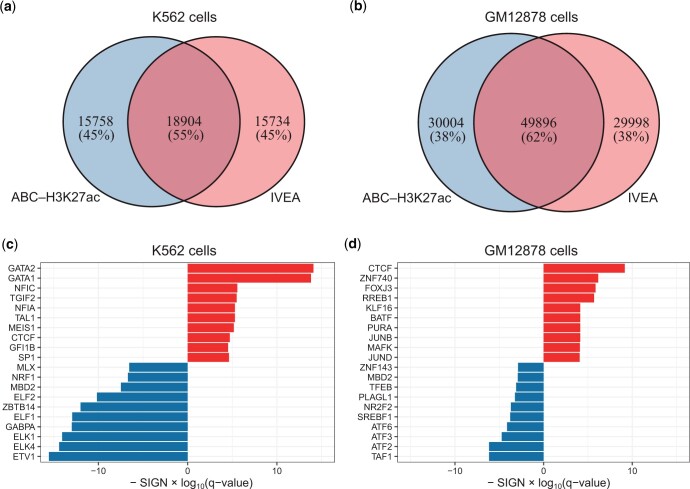
Characteristics of the predicted enhancer–gene interactions in the top-10-pair set predicted with cell-type-specific Hi-C. (a, b) Venn diagrams of the predicted enhancer–gene interactions from IVEA and ABC–H3K27ac in K562 (a) and GM12878 (b) cells. (c, d) Differentially enriched TF binding motifs detected in the predicted enhancers between IVEA and ABC–H3K27ac in K562 (c) and GM12878 (d) cells. SIGN takes +1 for overrepresented and −1 for underrepresented terms in IVEA.

The use of IVEA-estimated enhancer activities was found to result in higher prediction accuracy. To examine the impact of using IVEA-estimated enhancer activities, we compared the predictions of IVEA using an 8 TPM cut-off with those of the original ABC (ABC–H3K27ac), which uses the same scoring scheme except for enhancer activity. In the top-10-pair set, 45% and 38% of enhancer–gene interactions appeared exclusively in the IVEA or ABC–H3K27ac results in K562 and GM12878 cells with cell-type-specific Hi-C, respectively (Fig. 3a and b). To characterize these differences, we investigated the TF binding motifs in the enhancers. We detected TF footprints in the enhancer regions and identified TF motifs within the footprints. The number of TF motifs found in the top-10-pair set was then compared between IVEA and ABC–H3K27ac. In K562 cells, the TF binding motifs of GATA2 and GATA1 were enriched in IVEA (*q* < 1.6 × 10^−14^), whereas those of ETV1, ELK4, and ELK1 were enriched in ABC–H3K27ac (*q* < 8.8 × 10^−15^) (Fig. 3c). GATA1 and GATA2 are important TFs involved in the development and maintenance of the hematopoietic systems (Fujiwara 2017). ETV1, ELK4, and ELK1 are ubiquitously expressed across tissues and cell types; ELK1 and ELK4 act as cofactors of serum response factor (Cooper *et al.* 2007, Thiel *et al.* 2021). When comparing the top-10-pair set with the bottom 10 pairs, these TFs were not enriched in IVEA and ABC–H3K27ac, and a similar pattern was observed in the enrichment of TFs in both methods (Supplementary Fig. S6). This suggests that IVEA can capture enhancer regulation that is more specific to the K562 cell type. In GM12878 cells, the overall difference was smaller than that in K562 cells, but the TF binding motif of CTCF was enriched in IVEA (*q* < 7.0×10^−10^) whereas that of TAF1 was enriched in ABC (*q* < 7.0×10^−7^) (Fig. 3d). CTCF plays a critical role in the formation of chromatin domains and loops (Fudenberg *et al.* 2016, Nora *et al.* 2017), and its binding to enhancer elements facilitates enhancer–promoter interactions (Ren *et al.* 2017). Interestingly, the chromatin contact frequencies between CTCF binding site-containing enhancers and their target genes were markedly higher in IVEA than in ABC (Supplementary Fig. S7). TAF1 is a part of TFIID, a general TF involved in transcription initiation (Patel *et al.* 2020), and interacts with H3K27 acetylated nucleosomes (Gibbons *et al.* 2022). TAF1 enrichment may be related to an ABC feature that utilizes the H3K27ac signal.

### 3.6 Analysis of GWAS variants by predicted enhancer–gene interactions

Many disease-associated variants are located in non-coding loci and may affect enhancers; however, the target genes are often unclear. Using the NHGRI-EBI GWAS catalogue (Sollis *et al.* 2023), we investigated the autoimmune disease variants and regulated genes in predicted enhancer–gene interactions in GM12878 cells using GM12878 Hi-C. Among the enhancers from the top-10-pair set, 229 and 225 enhancers contained GWAS variants from IVEA and ABC–H3K27ac predictions, respectively. These enhancers were linked to 590 and 728 genes in the top-10-pair set from IVEA and ABC–H3K27ac, respectively. Similar tendencies were observed in gABC-scoring approaches. Specifically, gABC–IVEA detected 256 GWAS variants containing enhancers and 410 target genes, while gABC–H3K27ac detected 247 GWAS variants containing enhancers and 491 target genes. The results utilizing IVEA contained slightly more enhancers with GWAS variants, but fewer genes linked to them. Functions of the target genes were analysed using their Gene Ontology (GO) terms. The top 10 significantly enriched GO terms of biological processes were almost the same in the IVEA, ABC–H3K27ac, gABC–IVEA, and gABC–H3K27ac predictions and were related to immune response, cytokine-mediated signalling pathway, or defence response terms (Tables 4 and 5). Notably, however, the enrichments of all of these terms were higher in approaches that used IVEA-estimated enhancer activities (–IVEA) than in those using the geometric means of H3K27ac and Dnase-seq signals (–H3K27ac) (Tables 4 and 5). As a negative control, the bottom 10 pairs did not show significantly enriched GO terms related to the immune response (Supplementary Table S10). This finding suggests that predictions made using IVEA-estimated enhancer activities could prioritize more genes that are related to the immune response for the autoimmune GWAS variants.

**Table 4.** Gene Ontology (GO) enrichment analysis of target genes associated with enhancers containing variants identified through genome-wide association studies (GWAS) in the top-10-pair set predicted in GM12878 cells: comparison between IVEA and ABC–H3K27ac.^a^

GO term	Description	IVEA			ABC–H3K27ac		
		(229 enhancers—590 target genes)			(225 enhancers—728 target genes)		
		FDR *q*-value	Enrichment	Rank	FDR *q*-value	Enrichment	Rank
GO:0019221	Cytokine-mediated signalling pathway	1.60E−08	2.54	1	8.84E−07	2.23	1
GO:0002376	Immune system process	1.04E−08	1.73	2	9.81E−06	1.53	7
GO:0006955	Immune response	6.09E−08	2.30	3	7.25E−07	2.08	2
GO:0002684	Positive regulation of immune system process	1.34E−07	2.11	4	4.62E−06	1.89	4
GO:0002250	Adaptive immune response	1.15E−07	3.76	5	1.76E−06	3.25	3
GO:0050776	Regulation of immune response	1.48E−07	2.09	6	1.13E−05	1.85	6
GO:0006952	Defence response	3.86E−07	2.16	7	6.57E−04	1.78	11
GO:0002682	Regulation of immune system process	5.89E−07	1.82	8	7.73E−06	1.68	5
GO:0050778	Positive regulation of immune response	1.93E−06	2.19	9	4.22E−05	1.95	8
GO:0060333	Interferon-gamma-mediated signalling pathway	1.92E−06	4.96	10	6.46E−05	4.04	9

a The predictions were performed using the GM12878 Hi-C matrix.

**Table 5.** Gene Ontology (GO) enrichment analysis of target genes associated with enhancers containing variants identified through genome-wide association studies (GWAS) in the top-10-pair set predicted in GM12878 cells: comparison between gABC–IVEA and gABC–H3K27ac.^a^

GO term	Description	gABC–IVEA			gABC–H3K27ac		
		(256 enhancers—410 target genes)			(247 enhancers—491 target genes)		
		FDR *q*-value	Enrichment	Rank	FDR *q*-value	Enrichment	Rank
GO:0019221	Cytokine-mediated signalling pathway	5.79E−12	3.27	1	3.72E−13	3.12	1
GO:0002376	Immune system process	7.65E−11	2.00	2	3.12E−08	1.79	3
GO:0002682	Regulation of immune system process	8.79E−11	2.27	3	1.64E−07	1.95	9
GO:0006955	Immune response	1.58E−10	2.85	4	8.44E−09	2.54	2
GO:0002684	Positive regulation of immune system process	8.01E−10	2.55	5	1.25E−07	2.23	6
GO:0002250	Adaptive immune response	1.82E−09	4.90	6	1.12E−07	4.11	7
GO:0051249	Regulation of lymphocyte activation	1.80E−09	3.55	7	3.49E−05	2.68	14
GO:0050776	Regulation of immune response	2.52E−09	2.49	8	1.09E−07	2.24	4
GO:0007166	Cell surface receptor signalling pathway	2.64E−09	2.07	9	1.12E−07	1.89	8
GO:0002694	Regulation of leukocyte activation	4.75E−09	3.29	10	1.25E−05	2.62	12

a The predictions were performed using the GM12878 Hi-C matrix.

## 4 Discussion

In this study, we proposed a variational Bayesian inference method of RE activity, IVEA, for predicting enhancer–gene regulatory interactions. We modelled the gene regulatory mechanism of transcriptional bursting, characterized by burst size and frequency, which were represented by promoter and enhancer activities, respectively. Our results show that the predictions using IVEA-estimated enhancer activities can achieve a higher prediction accuracy and provide biologically relevant enhancer–gene regulatory interactions.

Recently, a new deep learning approach called Enformer has been developed. This approach utilizes a transformer-based model to predict gene expression and chromatin status based on DNA sequence (Avsec *et al.* 2021). By analysing the prediction process or DNA sequence mutagenesis, it is possible to identify enhancer–promoter regulations. However, the region that can be analysed is currently limited to <100 kb.

Another recent advancement in this field is STARE, which has refined the ABC score scheme to achieve higher prediction accuracy (Hecker *et al.* 2023). For gene-centric analysis, such as analysing the top 10 enhancers per gene in this study (Table 3), gABC may be more suitable due to its gene-specific relative enhancer activity calculated in the gABC score. In addition, IVEA has been shown to achieve higher accuracy by using Bayesian inference of enhancer activity. Furthermore, it has been found to have an additive effect on the performance of gABC and ABC_TSS (Table 3).

The high predictive performance of IVEA-estimated enhancer activities in predicting enhancer–gene regulatory interactions suggests that these estimated activities accurately reflect the regulatory potential to activate genes. Enhancer activity is a hypothetical value that is often measured by H3K27ac, H3K4me1, P300, and/or eRNA (Andersson and Sandelin 2020, Gasperini *et al.* 2020). These biochemical features are enriched in active enhancers but are not completely predictive of enhancer activity due to their diverse mechanisms (Beagrie and Pombo 2016, Gasperini *et al.* 2020). Since our model is based on gene regulatory mechanisms, other latent variables, such as promoter activity, also reflect some functional aspects of gene regulation and may be useful in other applications. As further characteristics of transcriptional regulatory mechanisms become available, it will be possible to update the model and improve its accuracy.

Filtering genes with low expression (<4 TPM) showed better predictive accuracy using IVEA (Table 1). Since an enhancer is a factor that activates genes, enhancers regulating lowly expressed genes may be weak, and their DHS peaks may not be detected. Furthermore, silencers may be involved in regulating lowly expressed genes. Recent studies have shown that silencers are often bifunctional, i.e. they can also act as enhancers, depending on the cellular context (Segert *et al.* 2021). There is no evidence of a chromatin signature common to all silencers (Pang and Snyder 2020, Segert *et al.* 2021), except for the open chromatin state. Since REs are based only on DHS peaks, the detected REs could contain silencers, and considering their repressive functionality in the model may improve the prediction accuracy.

There are two limitations that need to be addressed. First, to predict enhancer regulations for a specific gene, its promoter element needs to be detected, as our model considers promoter activity for the evaluation of gene expression [Equation (8)]. Since the promoter region of an expressed gene is open in general, it is detectable in most cases. However, if the position of the actual TSS is far from that in the gene annotation used for analysis, the promoter element cannot be detected. Accordingly, we must be careful about TSS positions. Second, the contribution of activity of each enhancer is considered to be additive in IVEA. Although most enhancers contribute linearly to gene expression, some genes are synergistically affected. Thus, exponential and logistic function models have been proposed (Dukler *et al.* 2016, Choi *et al.* 2021). By considering synergistic enhancer contributions, the prediction can be made more accurately. To achieve this, it is necessary to identify the enhancers that make synergistic contributions and the genes that are synergistically regulated. This knowledge will enable us to better elucidate enhancer–gene regulation.

## Supplementary Material

vbae118_Supplementary_Data

## Data Availability

The IVEA code is available on GitHub at https://github.com/yasumasak/ivea. The publicly available datasets used in this study are described in Supplementary Table S4.
