# Supplementary material for: IVEA: an integrative variational Bayesian inference method for predicting enhancer–gene regulatory interactions
Source: Bioinform Adv. 2024 Aug 20;4(1):vbae118. doi: 10.1093/bioadv/vbae118 (PMC11349192; doi:10.1093/bioadv/vbae118)
Supplement: vbae118_Supplementary_Data [file vbae118_supplementary_data.pdf]

Supplementary Figure S1

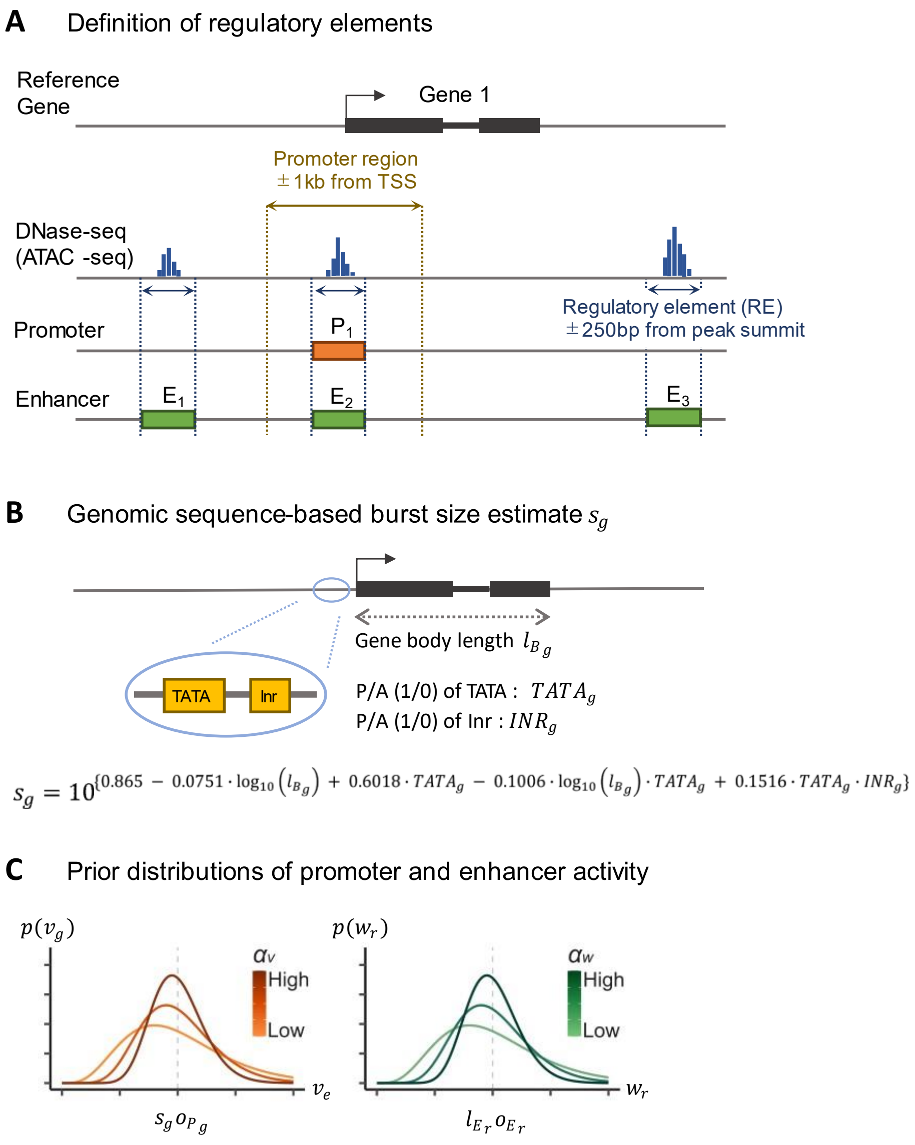

Figure S1. Schematic of the IVEA model. Related to Figure 1.

(A) Schematic diagram of promoter and enhancer elements defined by DNase-seq (or ATAC-seq) peaks and gene TSS positions. Regulatory elements (REs) were defined as ±250bp from the DNase-seq peak summit. The REs that were found at ±1kb from a gene TSS were considered promoter elements. All REs were considered enhancer elements.

(B) Schematic of genomic sequence-based burst size estimate s_g that were estimated by gene body length l_{B_g}, presence/absence of TATA $TATA_g$ and Inr INR_g.

(C) Schematic of prior distributions of promoter (left) and enhancer (right) activity, whose means are $s_g o_{P_g}$ and $l_{E_r} o_{E_r}$, respectively. Decrease of the shape parameter α_v and α_w broadens the prior distributions of the promoter and enhancer activity, respectively.

Supplementary Figure S2

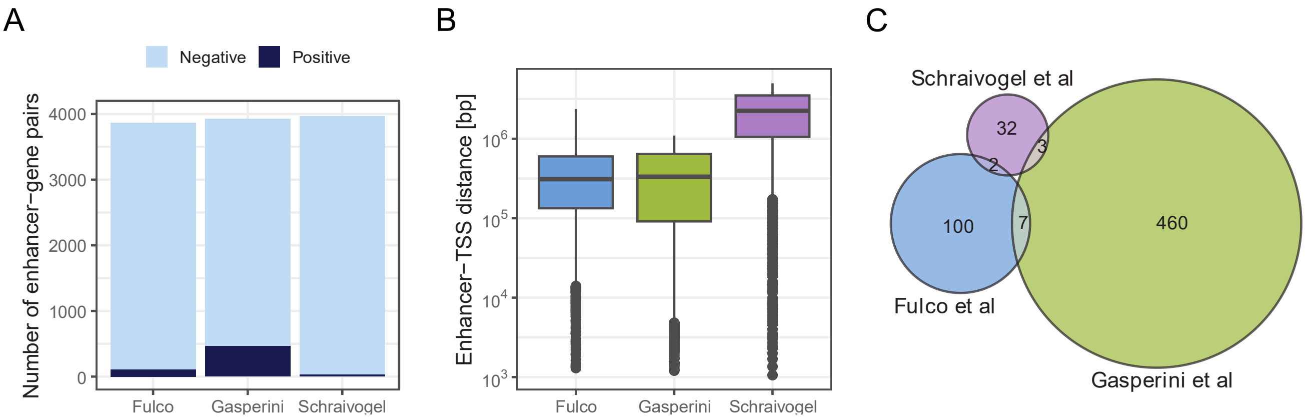

Figure S2. Statistics of enhancer–gene pairs in the three K562 CRISPRi datasets. The statistics were performed on the datasets subsequent to the extraction of positive and negative enhancer–gene pairs described in the Methods section. (A) Number of positive and negative enhancer–gene pairs in each dataset. (B) Distribution of enhancer–TSS distances within each dataset. (C) Intersection of positive enhancer–gene pairs across the three datasets.

Supplementary Figure S3

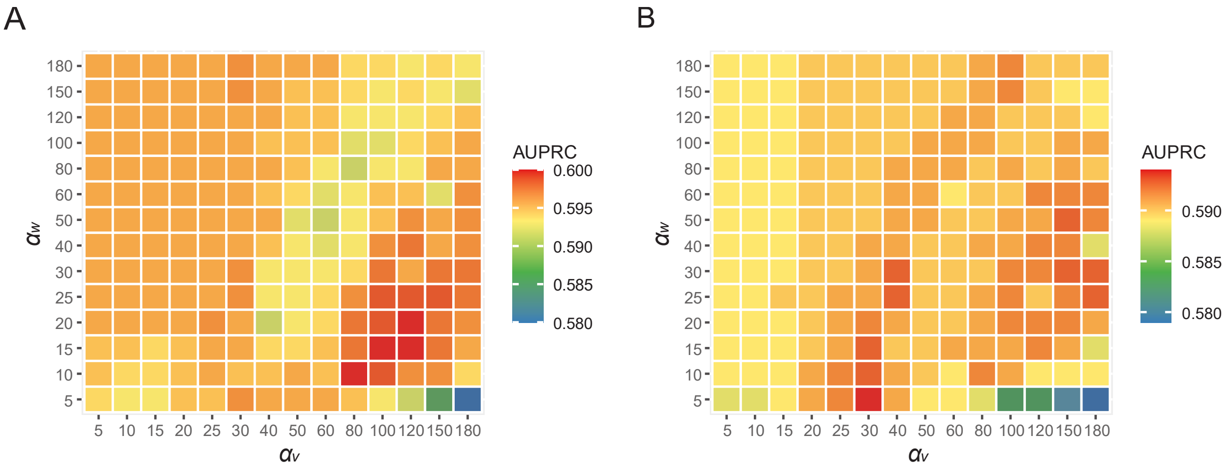

Figure S3. Effects of hyperparameters evaluated by CRISPRi perturbation data. AUPRCs of IVEA predictions with different hyperparameters α_v and α_w in the K562 training data using the K562 Hi-C (A) and the average Hi-C (B) matrix. IVEA was performed using an 8 TPM cut-off.

Related to Figure 2A.

Supplementary Figure S4

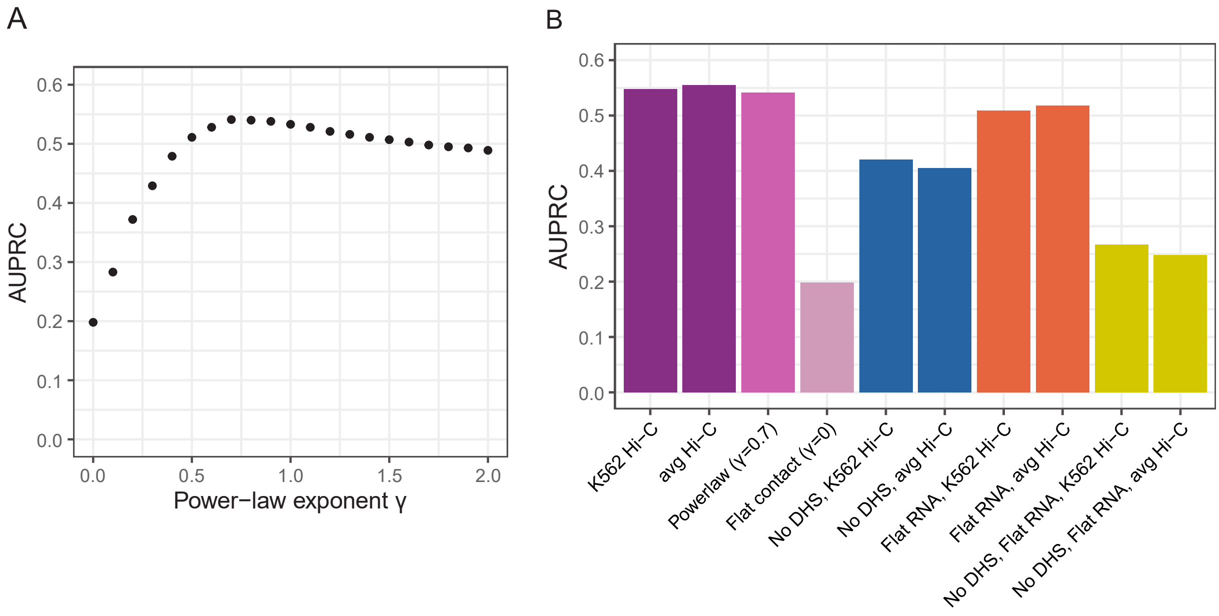

Figure S4. Effects of input data evaluated by K562 CRISPRi test data. IVEA was performed using an 8 TPM cut-off. (A) AUPRCs of IVEA predictions using chromatin contact frequencies approximated by a power-law function with different power-law exponents γ ($\text{Contact} \approx \text{Distance}^{-\gamma}$). A peak of the AUPRCs was found at γ = 0.7. (B) AUPRCs of IVEA with different settings of each input data in the K562 test data. 'K562 Hi-C', 'avg Hi-C', 'Powerlaw', and 'Flat contact' denote the use of the K562 Hi-C, the averaged Hi-C matrix, a power-law function of distance ($\text{Contact} \approx \text{Distance}^{-0.7}$), and a flat contact frequency ($\text{Contact} \approx 1$) over all distances for chromatin contact information, respectively. 'No DHS' refers to the exclusion of DHS values from the model, and 'Flat RNA' refers to the use of an artificial flat RNA-seq read count (1,000) across all genes. Applying both 'No DHS' and 'Flat RNA' means the use of Hi-C contact input only.

AUPRC, area under the precision–recall curve.

Supplementary Figure S5

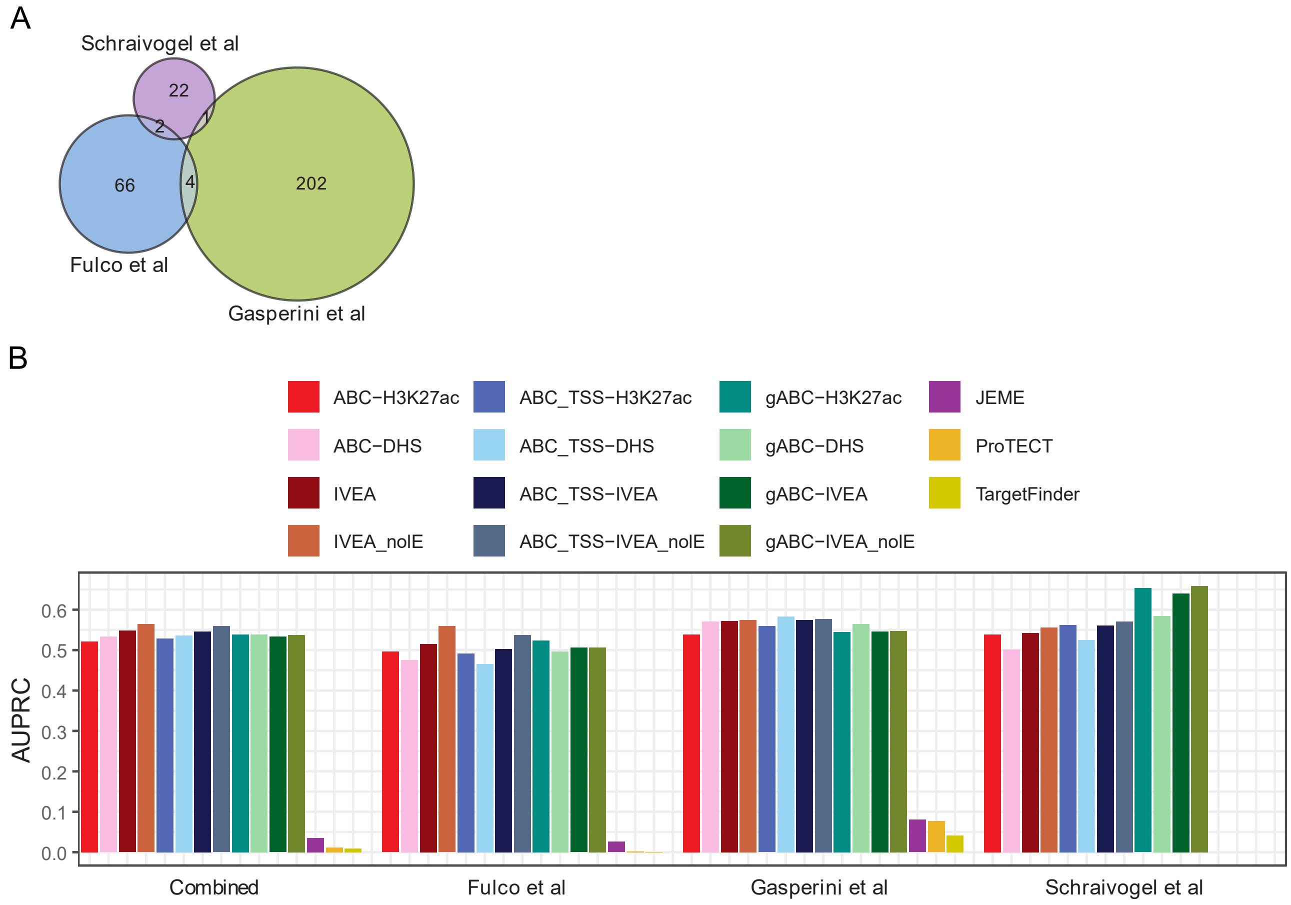

Figure S5. Comparison of the three studies used for the K562 CRISPRi test dataset. The CRISPRi-validated enhancer–gene pairs of genes with TPM > 8 were analysed. (A) Intersection of positive enhancer–gene pairs across the three studies. (B) AUPRCs of each method evaluated with each of the three CRISPRi studies, with the K562 Hi-C matrix used for chromatin contacts.

Supplementary Figure S6

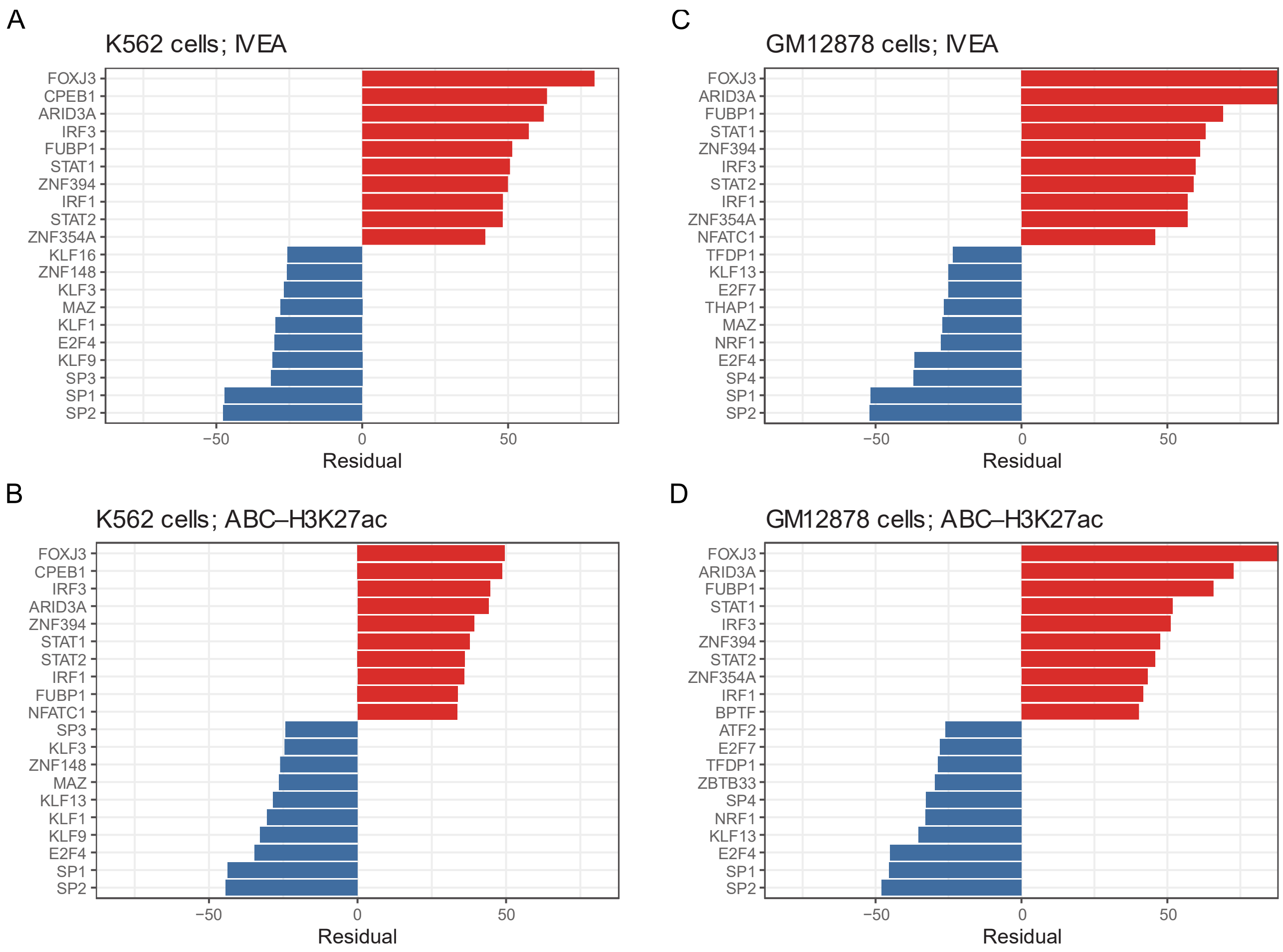

Figure S6. Differentially enriched TF binding motifs detected in the enhancers between the top-10-pair set and the bottom 10 pairs predicted with cell-type specific Hi-C. (A, B) Results from IVEA (A) and ABC–H3K27ac (B) in K562 cells. (C, D) Results from IVEA (C) and ABC–H3K27ac (D) in GM12878 cells. Residuals in the chi-square tests are shown as q-values are too small ($q < 10^{-140}$).

Related to Figure 3C and D.

Supplementary Figure S7

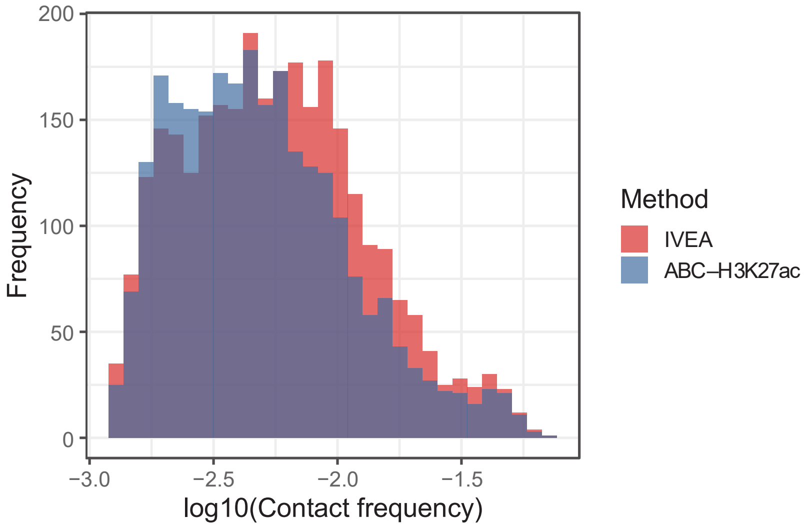

Figure S7. Chromatin contact frequencies between the CTCF binding site-containing enhancers and their target genes in the top-10-pair set (2900 and 2637 pairs from IVEA and ABC–H3K27ac, respectively). Higher contact frequencies were observed from IVEA than from ABC ($P < 1.1 \times 10^{-9}$, Mann–Whitney *U* test).

Related to Figure 3D.

Supplementary Note S1. Model omitting the relative enhancer element length l_{E_r} in the prior information on enhancer activity w_r (IVEA_nolE).

Omitting l_{E_r} in Equation (9) of the prior probability of enhancer activity w_r resulted in:

$$p(w_r|o_{E_r}) = \text{Gamma}\left(w_r \middle| \alpha_w, \frac{\alpha_w}{o_{E_r}}\right), \tag{9'}$$

This led to two parameter equations (Equations 26 and 31) in the variational Bayesian inference updated as:

$$\chi_{E_r} = 2\alpha_w \mathbb{E}_q[w_r], \tag{26'}$$

and

$$b_{w_r} = \alpha_w \mathbb{E}_q\left[o_{E_r}^{-1}\right] + \mathbb{E}_q[k] \sum_g l_{T_g} \mathbb{E}_q\left[v_g\right] c_{gr}. \tag{31'}$$

Please note that l_{E_r} remains in Equation (3) of DNase-seq (or ATAC-seq) counts on enhancer element and its related Equation (27) in the variational Bayesian inference.

The initial value of enhancer activity in the variational Bayesian inference was set as:

$$\mathbb{E}_q[w_r] = \left(\frac{x_{E_r}}{l_{E_r}} + 0.01\right).$$

Supplementary Table S1. AUPRCs of simpler IVEA models evaluated using the K562 CRISPRi test data

IVEA gene expression cut-off [TPM]	Not considering promoter activity	Not considering latent openness	Full IVEA model
cut-off 4 TPM	0.221	0.529	0.542
cut-off 8 TPM	0.22	0.54	0.548

AUPRC, area under the precision–recall curve; TPM, transcripts per million.
The predictions were made using the K562 Hi-C matrix.

The model not considering promoter activity is equivalent to omitting the promoter activity variable v_g and promoter-related variables x_{P_g}, o_{P_g}, s_g, and l_{P_g} in the full IVEA model. The model not considering latent openness variables directly uses observed DNase-seq read counts (x_{P_g} and x_{E_r} for promoter and enhancer elements, respectively) divided by their relative element lengths (l_{P_g} and l_{E_r} for promoter and enhancer elements, respectively) as openness values.

Supplementary Table S2. Hyperparameter values used in IVEA

Hyperparameter	Value
α_o	0.01
β_o	0.0001
α_k	0.001
β_k	0.001

Supplementary Table S3. Initial values used in IVEA

Expectation	Initial value
$\mathbb{E}_q[k]$	$\frac{2}{G}\sum_g \left\{ \frac{\frac{t_g}{l_{T_g}}}{s_g \frac{x_{P_g}}{l_{P_g}} \Sigma_r (c_{gr} x_{E_r})} \right\}$
$\mathbb{E}_q[o_{P_g}^{-1}]$	$\left(\frac{l_{P_g}}{x_{P_g} + 0.01} \right)$
$\mathbb{E}_q[o_{E_r}^{-1}]$	$\left(\frac{l_{E_r}}{x_{E_r} + 0.01} \right)$
$\mathbb{E}_q[v_g]$	$\left(x_{P_g} \frac{s_g}{l_{P_g}} + 0.01 \right)$
$\mathbb{E}_q[w_r]$	$(x_{E_r} + 0.01)$
$\mathbb{E}_q[\ln v_g]$	$\ln \left(x_{P_g} \frac{s_g}{l_{P_g}} + 0.01 \right)$
$\mathbb{E}_q[\ln w_r]$	$\ln(x_{E_r} + 0.01)$

Supplementary Table S4. Sources of the used data

Cell type	Assay	Source
K562	RNA-seq	ENCFF001REG.fastq.gz, ENCFF001REF.fastq.gz
K562	DNase-seq	wgEncodeUwDnaseK562AlnRep1.bam, wgEncodeUwDnaseK562AlnRep2.bam
K562	Hi-C	GSE63525_K562_combined_30.hic
K562	H3K27ac ChIP-seq	ENCFF384ZZM.bam
GM12878	RNA-seq	ENCFF001REI.fastq.gz, ENCFF001REH.fastq.gz
GM12878	DNase-seq	ENCFF775ZJX.bam, ENCFF783ZLL.bam
GM12878	Hi-C	GSE63525_GM12878_insitu_primary_30.hic
GM12878	H3K27ac ChIP-seq	ENCFF197QHX.bam, ENCFF882PRP.bam
Average of 10 cell lines	Hi-C	ftp://ftp.broadinstitute.org/outgoing/lincRNA/average_hic/average_hic.v2.191020.tar.gz

Supplementary Table S5. Training and test datasets of K562 cells separated by chromosomes

	Training	Test
Chromosomes	1, 2, 4, 5, 6, 8, 10, 16, 17, 20, 21, 22	3, 7, 9, 11, 12, 13, 14, 15, 18, 19, X
# Genes analysed	4214	3468
In CRISPRi validated pairs:		
# Genes	162	150
# Positive pairs	233	243
# Negative pairs	2605	6852

Supplementary Table S6. Number of analysed and evaluated genes in the K562 test datasets at different expression levels

	Expression levels of genes [TPM]						
	> 0	> 1	> 2	> 4	> 8	> 16	> 32
# Genes analysed	5943	4941	4584	4154	3468	2538	1644
In CRISPRi validated pairs:							
# Genes	183	176	168	162	150	128	96
# Positive pairs	285	278	269	262	243	219	178
# Negative pairs	7310	7266	7206	7170	6852	6450	5693

Supplementary Table S7. AUPRCs of IVEA_nolE with different expression cut-offs evaluated using the K562 CRISPRi test data

**IVEA_nolE gene expression cut-off [TPM]**	**Expression levels of evaluated genes [TPM]**						
	**>0**	**>1**	**>2**	**>4**	**>8**	**>16**	**>32**
IVEA_nolE cut-off 0	**0.53**	0.532	0.537	0.537	0.548	0.567	0.575
IVEA_nolE cut-off 1	-	**0.541**	0.546	0.546	0.555	0.575	0.584
IVEA_nolE cut-off 2	-	-	**0.55**	0.55	0.559	0.578	0.585
IVEA_nolE cut-off 4	-	-	-	**0.555**	**0.564**	0.583	0.591
IVEA_nolE cut-off 8	-	-	-	-	**0.564**	0.584	0.591
IVEA_nolE cut-off 16	-	-	-	-	-	**0.587**	**0.594**
IVEA_nolE cut-off 32	-	-	-	-	-	-	0.588

The highest AUPRC value within each column is highlighted in bold.
AUPRC, area under the precision–recall curve; TPM, transcripts per million.
The predictions were made using the K562 Hi-C matrix.

Supplementary Table S8. AUPRCs of IVEA considering all TSSs (IVEA_TSS) with different expression cut-offs evaluated using the K562 CRISPRi test data

**IVEA_TSS transcript expression cut-off [TPM]**	**Expression levels of evaluated genes [TPM]**					
	**> 1**	**> 2**	**> 4**	**> 8**	**> 16**	**> 32**
IVEA_TSS cut-off 0	**0.471**	0.474	0.469	0.479	0.492	0.493
IVEA_TSS cut-off 1	-	**0.508**	0.503	0.51	0.525	0.531
IVEA_TSS cut-off 2	-	-	**0.512**	0.519	0.535	0.537
IVEA_TSS cut-off 4	-	-	-	**0.526**	0.542	0.547
IVEA_TSS cut-off 8	-	-	-	-	**0.55**	**0.557**
IVEA_TSS cut-off 16	-	-	-	-	-	0.555

The highest AUPRC value within each column is highlighted in bold.
AUPRC, area under the precision–recall curve; TPM, transcripts per million.
The predictions were made using the K562 Hi-C matrix.

We examined an IVEA model that handles all TSSs (IVEA_TSS) by analysing all annotated transcriptional isoforms in the Gencode annotation (gencode.v26lift37.annotation.gtf). In the IVEA_TSS model, the gene g in the equations for gene-based IVEA was replaced by TSS u. The score for IVEA_TSS is calculated as its relative contribution to the expression of the gene and is written as:

$$h_{gr} = \frac{\sum_u v_u^* c_{ur} w_r^*}{\sum_{u'} v_{u'}^* \sum_{r'} \left(c_{ur'} \, w_{r'}^* \right)}. \quad (35)$$

Supplementary Table S9. Number of eQTL-supported enhancer–gene pairs in a set of the top 5 high-scored enhancer–gene pairs for each gene

Methods	K562					GM12878				
	Number of predicted pairs	Number of eQTL-supported pairs (% in predicted pairs)				Number of predicted pairs	Number of eQTL-supported pairs (% in predicted pairs)			
		GTEx Whole Blood		GEUVADIS LCLs			GTEx Whole Blood		GEUVADIS LCLs	
ABC–H3K27ac	17339	2138	(12.33%)	392	(2.26%)	39950	5527	(13.83%)	1227	(3.07%)
ABC–DHS	17339	2140	(12.34%)	394	(2.27%)	39950	5386	(13.48%)	1166	(2.92%)
IVEA	17328	2230	(12.87%)	412	(2.38%)	39950	5560	(13.92%)	1238	(3.10%)
IVEA_noIE	17328	2239	(12.92%)	423	(2.44%)	39950	5663	(14.18%)	1260	(3.15%)
ABC_TSS–H3K27ac	17310	2329	(13.45%)	440	(2.54%)	39935	6031	(15.10%)	1334	(3.34%)
ABC_TSS–DHS	17313	2308	(13.33%)	433	(2.50%)	39935	5940	(14.87%)	1293	(3.24%)
ABC_TSS–IVEA	17313	2418	(13.97%)	452	(2.61%)	39935	6159	(15.42%)	1384	(3.47%)
ABC_TSS–IVEA_noIE	17313	2431	(14.04%)	475	(2.74%)	39935	6240	(15.63%)	1383	(3.46%)
gABC–H3K27ac	17310	2842	(16.42%)	561	(3.24%)	39935	7151	(17.91%)	1610	(4.03%)
gABC–DHS	17313	2844	(16.43%)	555	(3.21%)	39935	7149	(17.90%)	1604	(4.02%)
gABC–IVEA	17313	**2883**	(16.65%)	**569**	(3.29%)	39935	**7204**	(18.04%)	**1624**	(4.07%)
gABC–IVEA_noIE	17313	2834	(16.37%)	568	(3.28%)	39935	7069	(17.70%)	1580	(3.96%)
JEME	10020	1550	(15.47%)	280	(2.79%)	21848	3551	(16.25%)	793	(3.63%)
ProTECT	11910	1177	(9.88%)	183	(1.54%)	22911	1514	(6.61%)	297	(1.30%)
TargetFinder	9551	184	(1.93%)	31	(0.32%)	22926	704	(3.07%)	139	(0.61%)

The highest number of pairs within each eQTL dataset and type of Hi-C data is highlighted in bold.
LCLs, lymphoblastoid cell lines.
The predictions were performed using the cell-type-specific Hi-C matrix.

Supplementary Table S10. Gene Ontology (GO) enrichment analysis of target genes associated with enhancers containing variants identified through genome-wide association studies (GWAS) in the bottom 10 pairs predicted by IVEA (A), ABC–H3K27ac (B), gABC–IVEA (C), and gABC–H3K27ac in GM12878 cells. The predictions were performed using the GM12878 Hi-C matrix.

A

		**IVEA (26 enhancers - 199 target genes)**		
**GO term**	**Description**	**FDR q-value**	**Enrichment**	**Rank**
GO:0006342	chromatin silencing	1.05E-01	6.45	1
GO:0019731	antibacterial humoral response	6.58E-02	15.36	2
GO:0006334	nucleosome assembly	1.23E-01	4.94	3
GO:0002227	innate immune response in mucosa	9.86E-02	19.11	4
GO:0002385	mucosal immune response	7.89E-02	19.11	5
GO:0045814	negative regulation of gene expression, epigenetic	1.10E-01	5.16	6
GO:0016458	gene silencing	1.14E-01	4.53	7
GO:0009617	response to bacterium	1.13E-01	4.96	8
GO:0016233	telomere capping	1.01E-01	10.75	9
GO:0002251	organ- or tissue-specific immune response	9.96E-02	15.64	10

B

		**ABC–H3K27ac (32 enhancers - 183 target genes)**		
**GO term**	**Description**	**FDR q-value**	**Enrichment**	**Rank**
GO:0006334	nucleosome assembly	4.65E-07	7.7	1
GO:0034728	nucleosome organisation	2.51E-07	6.63	2
GO:0071824	protein-DNA complex subunit organisation	8.94E-06	4.76	3
GO:0065004	protein-DNA complex assembly	1.02E-05	5.17	4
GO:0040029	regulation of gene expression, epigenetic	5.27E-05	4.62	5
GO:0000183	chromatin silencing at rDNA	4.60E-05	15.02	6
GO:0006335	DNA replication-dependent nucleosome assembly	3.94E-05	15.02	7
GO:0034723	DNA replication-dependent nucleosome organisation	3.45E-05	15.02	8
GO:0006342	chromatin silencing	3.27E-05	7.88	9
GO:0006333	chromatin assembly or disassembly	6.45E-05	6.66	10

C

GO term	Description	gABC–IVEA (59 enhancers - 185 target genes)		
		**FDR q-value**	**Enrichment**	**Rank**
GO:0002483	antigen processing and presentation of endogenous peptide antigen	2.05E-01	14.17	1
GO:0019883	antigen processing and presentation of endogenous antigen	2.76E-01	11.81	2
GO:0001916	positive regulation of T cell-mediated cytotoxicity	1.00E+00	11.34	3
GO:0006438	valyl-tRNA aminoacylation	1.00E+00	42.52	4
GO:0002491	antigen processing and presentation of endogenous peptide antigen via MHC class II	1.00E+00	42.52	5
GO:0002468	dendritic cell antigen processing and presentation	1.00E+00	42.52	6
GO:0002469	myeloid dendritic cell antigen processing and presentation	9.53E-01	42.52	7
GO:0071921	cohesin loading	8.34E-01	42.52	8
GO:0001914	regulation of T cell-mediated cytotoxicity	1.00E+00	8.95	9

D

GO term	Description	gABC–H3K27ac (49 enhancers - 172 target genes)		
		**FDR q-value**	**Enrichment**	**Rank**
GO:0043380	regulation of memory T cell differentiation	1.00E+00	27.86	1
GO:0043382	positive regulation of memory T cell differentiation	5.76E-01	27.86	2
GO:0045591	positive regulation of regulatory T cell differentiation	1.00E+00	19.9	3
GO:0002491	antigen processing and presentation of endogenous peptide antigen via MHC class II	1.00E+00	46.44	4
GO:0002468	dendritic cell antigen processing and presentation	1.00E+00	46.44	5
GO:0002469	myeloid dendritic cell antigen processing and presentation	9.32E-01	46.44	6
GO:0016233	telomere capping	1.00E+00	9.29	7
GO:0000183	chromatin silencing at rDNA	1.00E+00	8.84	8
GO:0006335	DNA replication-dependent nucleosome assembly	1.00E+00	8.84	9
GO:0034723	DNA replication-dependent nucleosome organisation	1.00E+00	8.84	10
